# Assessment of performance of the Gail model for predicting breast cancer risk: a systematic review and meta-analysis with trial sequential analysis

**DOI:** 10.1186/s13058-018-0947-5

**Published:** 2018-03-13

**Authors:** Xin Wang, Yubei Huang, Lian Li, Hongji Dai, Fengju Song, Kexin Chen

**Affiliations:** Department of Epidemiology and Biostatistics, Key Laboratory of Cancer Prevention and Therapy, Tianjin Key Laboratory of Breast Cancer Prevention and Therapy, Ministry of Education, National Clinical Research Center for Cancer, Tianjin Medical University Cancer Institute and Hospital, Huanhu Xi Road, Tiyuan Bei, Hexi District, Tianjin, 300060 People’s Republic of China

**Keywords:** Breast cancer, Gail model, Systematic review, Meta-analysis, Trial sequential analysis

## Abstract

**Background:**

The Gail model has been widely used and validated with conflicting results. The current study aims to evaluate the performance of different versions of the Gail model by means of systematic review and meta-analysis with trial sequential analysis (TSA).

**Methods:**

Three systematic review and meta-analyses were conducted. Pooled expected-to-observed (E/O) ratio and pooled area under the curve (AUC) were calculated using the DerSimonian and Laird random-effects model. Pooled sensitivity, specificity and diagnostic odds ratio were evaluated by bivariate mixed-effects model. TSA was also conducted to determine whether the evidence was sufficient and conclusive.

**Results:**

Gail model 1 accurately predicted breast cancer risk in American women (pooled E/O = 1.03; 95% CI 0.76–1.40). The pooled E/O ratios of Caucasian-American Gail model 2 in American, European and Asian women were 0.98 (95% CI 0.91–1.06), 1.07 (95% CI 0.66–1.74) and 2.29 (95% CI 1.95–2.68), respectively. Additionally, Asian-American Gail model 2 overestimated the risk for Asian women about two times (pooled E/O = 1.82; 95% CI 1.31–2.51). TSA showed that evidence in Asian women was sufficient; nonetheless, the results in American and European women need further verification.

The pooled AUCs for Gail model 1 in American and European women and Asian females were 0.55 (95% CI 0.53–0.56) and 0.75 (95% CI 0.63–0.88), respectively, and the pooled AUCs of Caucasian-American Gail model 2 for American, Asian and European females were 0.61 (95% CI 0.59–0.63), 0.55 (95% CI 0.52–0.58) and 0.58 (95% CI 0.55–0.62), respectively.

The pooled sensitivity, specificity and diagnostic odds ratio of Gail model 1 were 0.63 (95% CI 0.27–0.89), 0.91 (95% CI 0.87–0.94) and 17.38 (95% CI 2.66–113.70), respectively, and the corresponding indexes of Gail model 2 were 0.35 (95% CI 0.17–0.59), 0.86 (95% CI 0.76–0.92) and 3.38 (95% CI 1.40–8.17), respectively.

**Conclusions:**

The Gail model was more accurate in predicting the incidence of breast cancer in American and European females, while far less useful for individual-level risk prediction. Moreover, the Gail model may overestimate the risk in Asian women and the results were further validated by TSA, which is an addition to the three previous systematic review and meta-analyses.

**Trial registration:**

PROSPERO CRD42016047215.

**Electronic supplementary material:**

The online version of this article (10.1186/s13058-018-0947-5) contains supplementary material, which is available to authorized users.

## Background

Breast cancer is the most common cancer in women with high morbidity and mortality rates [1]. Risk assessment tools estimating the individual’s absolute risk for developing breast cancer and identifying the women at high level of risk are crucial for decision-making about prevention and screening.

The Breast Cancer Risk Assessment Tool (BCRAT) [2], also known as the Gail model, was the most widely used appraisal tool for predicting the absolute risk of developing breast cancer. Individuals with 5-year risk exceeding 1.67% were considered high risk [3]. In 1992, the tool was modified to specifically predict invasive breast cancer, and this updated model, referred to as Gail model 2 (Caucasian-American Gail model) [4], has been used for determining the eligibility of subjects for chemoprevention of invasive breast cancer [5, 6]. In addition, this modified Gail model was also updated subsequently to predict the risk for other ethnic populations, such as African-American [7] and Asian-American [8] females.

A number of studies have been conducted to validate the Gail model in American [9–27], European [28–37], Asian [38–50] and Oceanian [51, 52] women. However, these studies showed variability in their calibration (expected-to-observed (E/O) ratio) and discrimination (Concordance-statistic (C-statistic) or area under the curve (AUC)). Although three systematic review and meta-analyses validated the Gail model previously [53–55], 19 studies [13, 14, 17–20, 22–24, 32–36, 38, 40, 41, 51, 52] with inconsistent results have been published subsequently or were not included in the previous meta-analyses. However, the evaluation studies launched in China [39, 42–50] have not been incorporated before and the diagnostic accuracy of the Gail model has not been fully evaluated.

There is increasing awareness that a meta-analysis also needs sufficient sample size to get a stable conclusion. Trial sequential analysis (TSA) was introduced to calculate the required information size (RIS) for meta-analysis and to decide whether the evidence was sufficient and conclusive [56, 57].

Here, we conducted a systematic review and meta-analysis to comprehensively evaluate the performance of different versions of the Gail model from three different dimensions (calibration, discrimination and diagnostic accuracy). In addition, the meta-analysis for calibration of the Gail model was also challenged by TSA.

## Methods

### Study registration

The current systematic review and meta-analysis was performed according to MOOSE guidelines [58] and has been registered with the International Prospective Register of Systematic Reviews (PROSPERO; registration number CRD42016047215).

### Literature search strategy

Two investigators conducted a literature search in the PubMed, Embase, WANFANG [59], VIP [60] and China National Knowledge Infrastructure (CNKI) [61] databases for all articles concerning the performance of the Gail model in females.

We used “mammary OR breast cancer OR carcinoma OR tumor OR neoplasm” AND “calibration OR validate OR validation OR screen OR screening OR expected-to-observed ratio OR E/O ratio” AND “Gail model OR breast cancer risk assessment tool OR BCRAT” as medical subject headings (MeSH) in searching for studies evaluating the calibration of the Gail model.

The terms “mammary OR breast cancer OR carcinoma OR tumor OR neoplasm” AND “discrimination OR validate OR validation OR screen OR screening OR sensitivity OR specificity OR area under the curve OR AUC OR C-statistic” AND “Gail model OR breast cancer risk assessment tool OR BCRAT” were used for retrieving publications assessing the discrimination and diagnostic accuracy of the Gail model.

Publications in English and Chinese language between 1 January 1989 (when the Gail model was developed [3]) and 31 July 2016 were included. Listed references were also manually checked for relevant papers.

### Inclusion and exclusion criteria

The inclusion and exclusion criteria for this meta-analysis included the following: studies validating the performance of the original (Gail model 1) or modified (Gail model 2) Gail model in women [3, 4]; calibration of the Gail model was prospectively estimated focusing on cohort studies that provided the E/O ratio and its 95% confidence interval (CI) or offered sufficient data for calculating the expected and observed number of breast cancer; discrimination of the Gail model was estimated focusing on the studies providing the C-statistic or AUC and its 95% CI for the Gail model; the diagnostic meta-analysis included publications that provided sufficient data for calculating the true positive (TP), false positive (FP), false negative (FN) and true negative (TN) values of the Gail model, respectively; the threshold of the Gail model was limited to ≥ 1.67%; the sample size should be higher than 100 and the mean follow-up period for the cohort studies should be longer than 1 year; and when multiple publications included the same population, studies with larger sample size or longer follow-up period were incorporated and studies with independent validations in subsequent articles were included.

### Literature selection for the systematic review and meta-analysis

For the calibration of the Gail model, 435 studies were found in the electronic databases and 10 were manually retrieved. After careful examination, 419 publications were excluded: 62 were duplicated records, 235 were not related, 70 were reviews and 52 were conference abstracts. In addition, two studies were excluded [27, 62] as they focused on the same population but with smaller sample size than other studies [17, 31]. In the end, 24 studies with 29 datasets were included.

After excluding the duplicated records, 356 studies were retrieved for estimating the discrimination of the Gail model. Of these, 311 were excluded in the preliminary screening and 19 were further eliminated by full-text reading. Moreover, seven studies [31, 62–67] were also excluded as they focused on the same population but with a shorter study period or smaller sample size than other included studies [17, 27, 51]. In total, 26 studies incorporating 29 datasets were included in this meta-analysis.

**Fig. 1:**
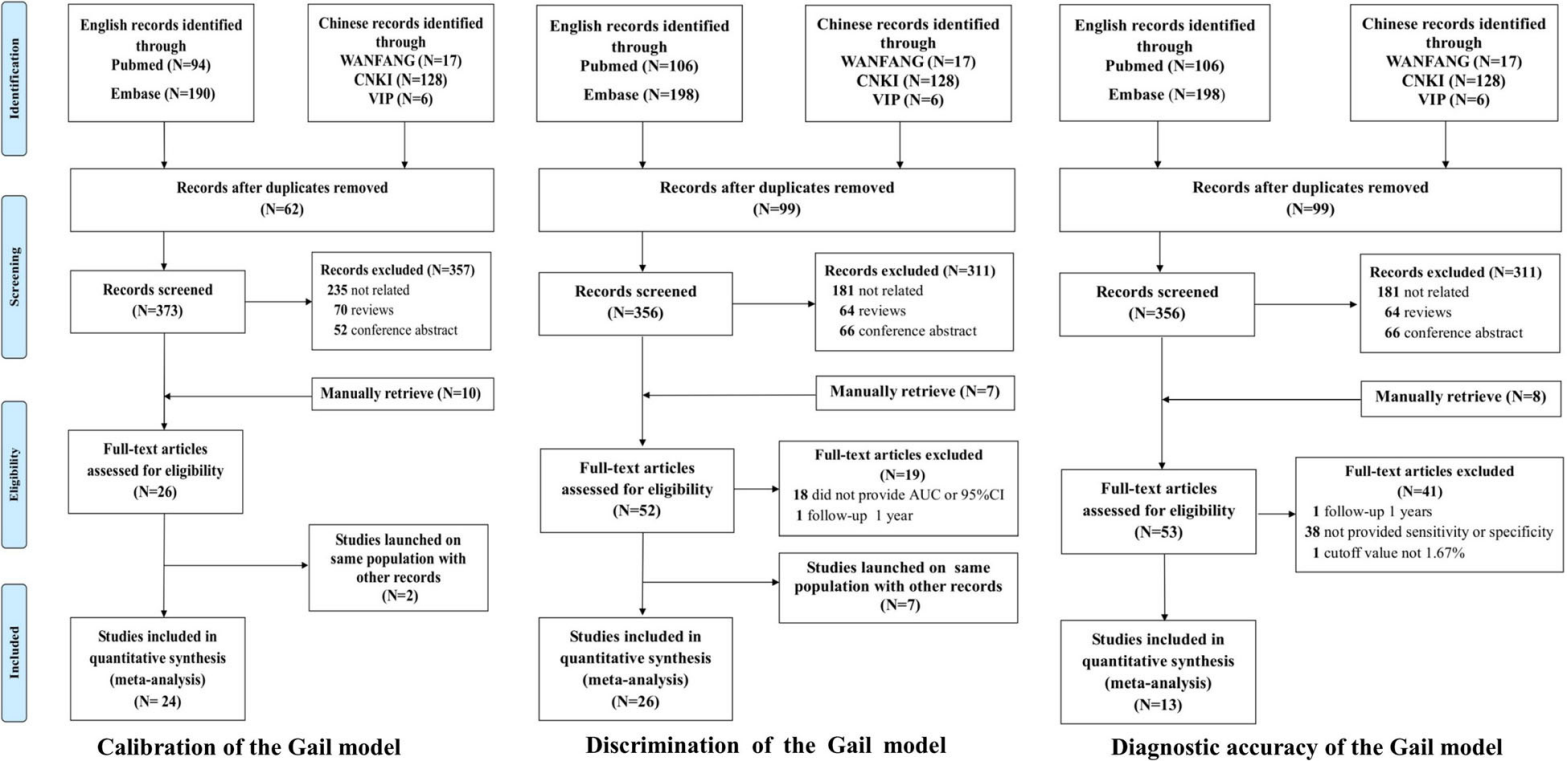
Flowchart of study selection in the meta-analyses for estimating the calibration, discrimination and diagnostic accuracy of the Gail model. AUC area under the curve, CI confidence interval, CNKI China National Knowledge Infrastructure

For the diagnostic accuracy of the Gail model, 455 publications were retrieved at the beginning. After preliminary screening and the full-text reading, 13 studies were finally included (Fig. 1).

Studies included in the aforementioned three meta-analyses overlapped to some extent, as some of them provided both the E/O ratio and AUC or the diagnostic accuracy of the Gail model [11, 15, 17, 18, 20, 30–33, 35, 39, 41, 44, 45].

### Data abstraction

Two investigators independently extracted data. Relevant information included the first author, publication year, geographic region, versions of the Gail model (Gail model 1 or Gail model 2 for Caucasian-American, Asian-American and African-American women), risk prediction period, study design, study population, sample size, mean age of participants as well as the risk for breast cancer, study period, follow-up period, E/O ratio with 95% CI, C-statistic or AUC with 95% CI and number of true positive (TP), false positive (FP), false negative (FN) and true negative (TN) values. The quality of the included studies was assessed by Newcastle–Ottawa Scale (NOS) [68] and the studies incorporated in the diagnostic meta-analysis were assessed by Quality Assessment Diagnostic Accuracy Studies (QUADAS) [69]. Any discrepancies were resolved by consensus, and where needed the corresponding author was contacted.

#### Statistical analyses

Calibration assessed how closely the number of subjects predicted to develop breast cancer matched the observed number of breast cancer cases diagnosed during a specific period. This was calculated by E/O ratio and the 95% CI of the E/O ratio was computed as: E/O ratio × exp.(± 1.96 × 1/√(O)) [11]. A well-fitting calibration should be close to 1.0. The discrimination value was assessed by C-statistic, which measures the Gail model’s ability to discriminate the women who will and will not develop breast cancer; moreover, it was considered identical to the AUC in the current study [54]. A C-statistic/AUC of 0.5 was considered as no discrimination, whereas 1.0 indicates perfect discrimination.

The pooled E/O ratio and C-statistic/AUC of the Gail model were calculated using DerSimonian and Laird’s random-effects model [70]. The *I*^2^ value was employed to evaluate the heterogeneity among the studies, and subgroup analyses were carried out to identify the source of the heterogeneity. Sensitivity analysis was conducted to assess the influence of each study on the combined effects by sequentially omitting each dataset [71]. Cumulative meta-analyses were launched to investigate the trend of the pooled E/O ratio and C-statistic/AUC ranked by the publication year and sample size [72]. Visualized asymmetry of the funnel plot and Egger’s regression test were assessed to detect publication bias. Pooled effects were also adjusted by the Duval and Tweedie trim-and-fill method [73–75].

The pooled estimations of sensitivity, specificity and diagnostic odds ratio (DOR) were calculated using a bivariate mixed-effects model. The DOR is the ratio of risk odds in breast cancer cases relative to that in controls [76]. Publication bias was detected by Deeks’ funnel plot, using 1/root (effective sample size) vs log DOR. *P* < 0.05 for the slope coefficient indicates significant asymmetry [77].

In the current study, TSA was conducted to determine whether the sample size incorporated in the meta-analysis was sufficient for evaluating the calibration of the Gail model. The included cohort studies are identified as trials for calculating the difference in breast cancer incidence between the expected and observed groups, and accordingly the total sample size is doubled. For the TSA, when the *Z*-curve crosses the conventional boundary, a significant difference is considered to exist between the expected group and the observed group for breast cancer incidence. Moreover, if the *Z*-curve passes through the trial sequential monitoring boundary or required information size (RIS) boundary, the evidence is considered sufficient and conclusive. Otherwise, the evidence is adjudged inconclusive and more studies were required to further verify the results [56, 57]. Furthermore, in order to evaluate the effect of the Chinese studies on the performance of the Gail model, a sensitivity analysis was conducted by eliminating the studies retrieved from the WANFANG, VIP and CNKI databases.

Pooled E/O ratio and AUC were synthesized using Comprehensive Meta Analysis version 2.0 (Biostat, Englewood, NJ, USA). Pooled sensitivity, specificity and DOR were conducted with Stata statistical software version 14.0 (StataCorp, College Station, TX, USA). The trial sequential analyses program (version 0.9 beta) was used for the TSA [78] (Copenhagen Trial Unit, Centre for Clinical Intervention Research, Copenhagen, Denmark, 2011).

## Results

### Calibration of the Gail model

**Fig. 2:**
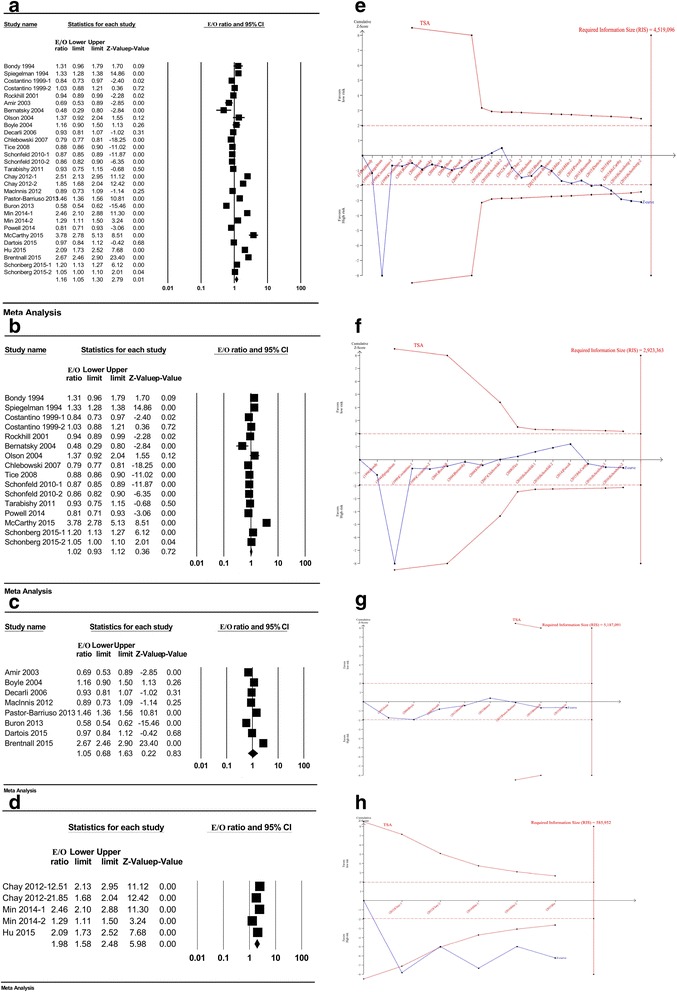
Calibration of the Gail model in total and stratified by geographic region with the trial sequential analysis. Forest plot of the pooled E/O ratio for the Gail model in total (**a**) and studies from America (**b**), Europe (**c**) and Asia (**d**), respectively. Trial sequential analysis (TSA) for pooled E/O ratio in total (**e**) and studies from America (**f**), Europe (**g**) and Asia (**g**), respectively. E/O expected-to-observed ratio, CI confidence interval

Twenty-four studies incorporating 29 records were included to evaluate the calibration of the Gail model [9–20, 28–35, 38, 39, 41, 52] (Table 1). The pooled E/O ratio was 1.16 (95% CI 1.05–1.30) with a high level of heterogeneity between studies (*I*^2^ = 98.8%; *p* < 0.01) (Fig. 2a). Sensitivity analysis showed that the combined E/O ratio and 95% CI were not significantly altered before and after the omission of each dataset (see Additional file 1A). Cumulative analysis showed that by continually increasing the publication year and the sample size, the 95% CI became narrower and the pooled E/O ratio was closer to 1.0, which indicates that the precision of the pooled E/O ratio was gradually improved (see Additional file 1B, C). Publication bias was detected by funnel plot (regression coefficient = 5.38; *p* = 0.027) (see Additional file 2A). According to the trim-and-fill method, the adjusted pooled E/O ratio was 1.25 (95% CI 1.11–1.40) after trimming (see Additional file 2B).

**Table 1:** Characteristics of the included studies for estimating the calibration of the Gail model

Reference	Author	Publication year	Geographic background	Gail model version	5/10-year risk	Sample size	Mean age (years)	Study population	Risk for breast cancer^a^	Time period	Follow-up period	E/O (95% CI)
[9]	Bondy	1994	America	1	5	1981	30–75	American Cancer Society 1987 Texas Breast Screening Project (with family history of breast cancer)	High risk	1987–1992	5.0	1.31 (0.96–1.79)
[10]	Spiegelman	1994	America	1	5	115,172	29–61	Nurses’ Health Study (NHS)	General population	1976–1981	6.0	1.33 (1.28–1.39)
[12]	Costantino-1	1999	America	1	5	5969	> 35	Placebo group of Breast cancer prevention trial (BCPT)	General population	1992–1998	4.03 (0.1–5.83)	0.84 (0.73–0.97)
[12]	Costantino-2	1999	America	2	5	5969	> 35	Placebo group of Breast cancer prevention trial (BCPT)	General population	1992–1998	4.03 (0.1–5.83)	1.03 (0.88–1.21)
[11]	Rockhill	2001	America	2	5	82,109	45–71	Nurses’ Health Study (NHS)	General population	1992–1997	5.0	0.94 (0.89–0.99)
[30]	Amir	2003	United Kingdom	2	10	3150	44 (21–73)	Women attending the Family History Screening Programme in University Hospital of South Manchester	Not defined	1987–2001	5.27 (0.1–15)	0.69 (0.54–0.90)
[13]	Bernatsky	2004	America	1	5	871	41 ± 13	Systemic lupus erythematosus clinic cohorts at Canada, Northwestern and UK center	High risk	1984–2000	9.1	0.48 (0.29–0.80)
[14]	Olson	2004	America	1	5	674	31–90	Women with possible bilateral oophorectomy identified from the Mayo Clinic Surgical Index	Low risk	1994–2004	NA	1.37 (0.92–2.04)
[28]	Boyle	2004	Italy	2	5	5383	NA	Women participated in RCT of tamoxifen for breast cancer prevention in Italy	General population	1992–2001	5.0	1.16 (0.89–1.49)
[29]	Decarli	2006	Italy	2	5	10,031	35–64	Florence—European Prospective Investigation Into Cancer and Nutrition Cohort (EPIC)	General population	1993–2002	9.0	0.93 (0.81–1.07)
[31]	Chlebowski	2007	America	2	5	147,916	63 (50–79)	Women’s Health Initiative (WHI)	General population	1993–2005	5.0	0.79 (0.77–0.82)
[15]	Tice	2008	America	2	5	629,229	40–74	National Cancer Institute-funded Breast Cancer Surveillance Consortium (BCSC)	General population	since 1994	5.3	0.88 (0.86–0.90)
[16]	Schonfeld-1	2010	America	2	5	181,979	62.8	National Institutes of Health-American Association of Retired Persons (NIH-AARP)	General population	1995–2003	7.5	0.87 (0.85–0.89)
[16]	Schonfeld-2	2010	America	2	5	64,868	62.3	Prostate, Lung, Colorectal and Ovarian Cancer Screening Trial (PLCO)	General population	1993–2006	8.6	0.86 (0.82–0.90)
[17]	Tarabishy	2011	America	2	5	4726	18–85	Mayo Benign Breast Disease (BBD)	High risk	1991–1996	5.0	1.08 (0.88–1.33)
[38]	Chay-1	2012	Singapore	3	5	28,104	50–64	Singapore Breast Cancer Screening Project (SBCSP)	General population	1997–2007	5.0	2.51 (2.14–2.96)
[38]	Chay-2	2012	Singapore	3	10	28,104	50–64	Singapore Breast Cancer Screening Project (SBCSP)	General population	1997–2007	10.0	1.85 (1.68–2.04)
[52]	Maclnnis	2012	Australia	NA	NA	2000	NA	Female relatives of the breast cancer cases in Australia	High risk	NA	10.0	0.89 (0.73–1.09)
[32]	Pastor-Barriuso	2013	Spain	2	5	54,649	45–68	Population-based Navarre Breast Cancer Screening Program (NBCSP)	General population	1996–2005	7.7	1.46 (1.36–1.56)
[33]	Buron	2013	Spain	2	5	2200	49–64	Participants with a positive screening mammogram in “Parc de Salut Mar” breast cancer screening program	High risk	2003–2010	6.0	0.58 (0.54–0.63)
[41]	Min-1	2014	Korea	2	5	40,229	> 10	Women routinely screened in Women’s Healthcare Center of Cheil General Hospital	Not defined	1999–2004	5.0	2.46 (2.10–2.87)
[41]	Min-2	2014	Korea	3	5	40,229	> 10	Women routinely screened in Women’s Healthcare Center of Cheil General Hospital	Not defined	1999–2004	5.0	1.29 (1.11–1.51)
[18]	Powell	2014	America	2	5	12,843	NA	Marin Women’s Study with high rate of breast cancer, null parity and delayed childbirth	High risk	2003–2007	5.0	0.81 (0.71–0.93)
[19]	McCarthy	2015	America	2	5	464	48.7 ± 13.2	Women referred for biopsy with abnormal (Breast Imaging Reporting And Data System, BI-RADS 4) mammograms at the Hospital of the University of Pennsylvania	High risk	2003–2012	5.0	3.78 (2.78–5.13)
[34]	Dartois	2015	France	2	5	13,174	42–72	Women in French E3N prospective cohort to investigate the cancer risk factors	General population	1993–1998	5.0	0.97 (0.84–1.12)
[39]	Hu	2015	China	2	5	42,908	35–69	Women participated in the breast cancer screening in Zhejiang eastern coastal areas of China	General population	2008–2014	5.0	2.09 (1.73–2.52)
[20]	Schonberg-1	2015	America	2	5	71,293	70 ± 7.0	Nurses’ Health Study (NHS)	High risk	2004–2009	5.0	1.20 (1.13–1.26)
[20]	Schonberg-2	2015	America	2	5	79,611	71 ± 6.8	Women’s Health Initiative (WHI), extensive study	High risk	2005–2010	5.0	1.05 (1.00–1.10)
[35]	Brentnall	2015	United Kingdom	2	10	50,628	47–73	15 screening areas in Greater Manchester, UK	General population	2009–2014	3.2	2.67 (2.46–2.90)

Note: Gail model type 1, original Gail model; Gail model type 2, modified Gail model for Caucasian-American; Gail model type 3, modified Gail model for Asian-American

*NA* not available, *E/O* expected-to-observed ratio, *CI* confidence interval, *RCT* randomized controlled trial

^a^Cohort studies enrolled women with high risk for breast cancer (with higher average age (> 70 years), dense mammary image, postmenopausal state, breast cancer relatives or high rate of delayed childbirth) were defined as “High risk”; cohort studies that did not accurately depict the characteristics of the participants were defined as “Not defined”. Participants with protective factors for breast cancer were considered low risk

Subgroup analysis suggested the geographic region (see Additional file 3) could partly explain the heterogeneities between these studies (*p* < 0.01). The Gail model exhibited a tendency to overpredict breast cancer risk for Asian women (pooled E/O = 1.98; 95% CI 1.58–2.48) compared to American (pooled E/O = 1.02; 95% CI 0.93–1.12) and European (pooled E/O = 1.05; 95% CI 0.68–1.63) women (Fig. 2b–d). Publication bias did not exist in each of these subgroups (see Additional file 4).

In addition, results showed that Gail model 1 accurately predicted breast cancer risk in American women (pooled E/O = 1.03; 95% CI 0.76–1.40). However, Gail model 2 overpredicted the risk for breast cancer (pooled E/O = 1.20; 95% CI 1.07–1.35) (see Additional file 3). When further stratified by different versions of Gail model 2, the pooled E/O ratios of Caucasian-American Gail model 2 in American [11, 12, 15–20, 31], European [28–30, 32–35] and Asian [39, 41] women were 0.98 (95% CI 0.91–1.06), 1.07 (95% CI 0.66–1.74) and 2.29 (95% CI 1.95–2.68), respectively. The pooled E/O ratio for Asian women was significantly higher than that in American and European females (*p* < 0.001). Moreover, only two studies clearly stated that they used the Asian-American Gail model [38, 41], and the results indicated that it overestimated the risk for Asian women about two times (pooled E/O = 1.82; 95% CI 1.31–2.51) (see Additional file 5).

When excluding studies conducted in Asian women [38, 39, 41], results showed that the Gail model precisely predicted the risk for developing breast cancer (pooled E/O = 1.04; 95% CI 0.93–1.16) (see Additional file 6A). Sensitivity analysis by singly eliminating each study showed no significant fluctuation, which indicated the stability of the results (see Additional file 6B). Cumulative analysis showed that the pooled E/O ratio became progressively closer to 1.0 according to accumulation of the publication year and sample size (see Additional file 6C, D). When stratified by different versions of the Gail model, the combined E/O ratios of Gail model 1 and Caucasian-American Gail model 2 were reported to be 1.03 (95% CI 0.76–1.40) and 1.05 (95% CI 0.93–1.17), respectively, with no significant difference (*p* = 0.93) (see Additional file 7). Stratified analysis showed that the studies with high reporting quality were prone to have a precise estimate of breast cancer risk (pooled E/O = 0.88; 95% CI 0.71–1.10 vs pooled E/O = 1.13; 95% CI 1.00–1.29; *p* = 0.06). However, no difference was found when stratified by the geographic region and other factors (see Additional file 8).

#### Trial sequential analysis

In the TSA, the cumulative *Z*-curve passed through both the conventional and the trial sequential monitoring boundary, which suggested the evidence was sufficient to verify the overprediction of the Gail model (Fig. 2e). When stratified by geographic region, the cumulative *Z*-curve did not cross the conventional and RIS boundary in American (Fig. 2f) and European (Fig. 2g) studies, demonstrating the accurate prediction of the Gail model. However, the evidence was insufficient to draw a firm conclusion and more related studies were required to confirm the results. With respect to Asian women, the *Z*-score crossed both the conventional and TSA-adjusted boundary, which showed the overestimation of breast cancer risk in Asian females and the evidence was sufficient and conclusive (Fig. 2h).

### Discrimination of the Gail model

**Fig. 3:**
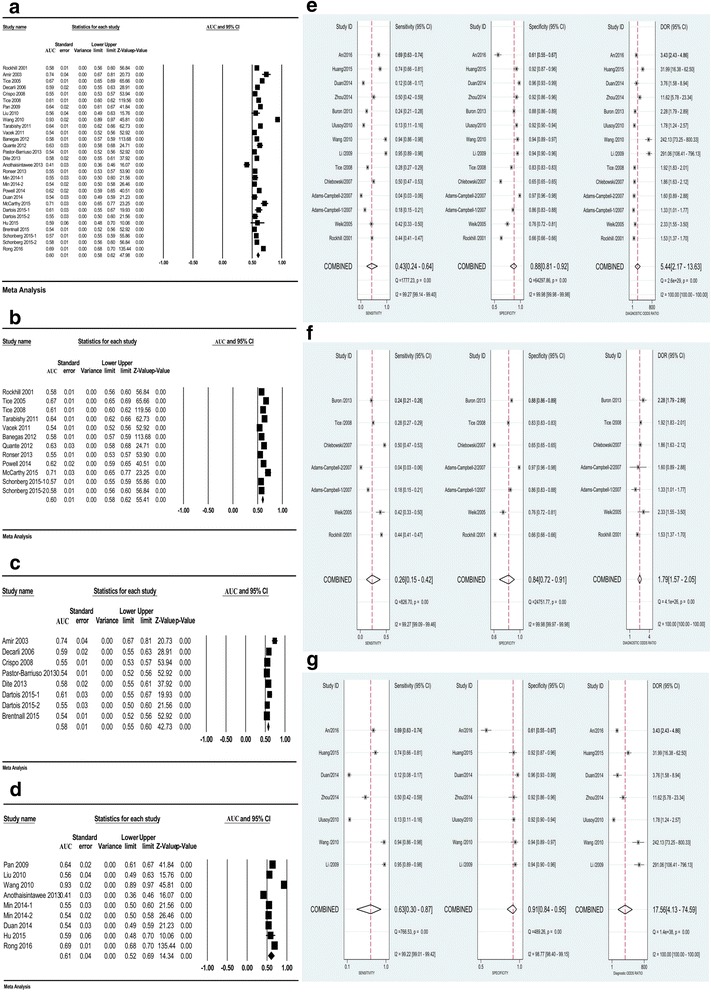
Pooled discrimination and diagnostic accuracy of the Gail model in total or stratified by geographic region. Pooled AUC/C-statistic of the Gail model in total (**a**) and studies from America (**b**), Europe (**c**) and Asia (**d**), respectively. Pooled sensitivity, specificity and diagnostic odds ratio (DOR) of the Gail model in total (**e**) and studies from America and Europe (**f**) and Asia (**g**), respectively. AUC area under the curve, CI confidence interval

Twenty-six articles with 29 datasets describing the C-statistic/AUC of the Gail model were combined to evaluate its pooled discrimination [11, 15, 18–24, 27, 29–32, 34–36, 39–46, 51] (Table 2). The pooled AUC was 0.60 (95% CI 0.58–0.62) with substantial heterogeneity (*I*^2^ = 97.0%; *p* < 0.01) (Fig. 3a). Sensitivity analysis suggested that the results were stable, and cumulative analysis indicated that the 95% CI became narrower and the pooled AUC progressively rose toward 0.60 with the accumulation of data ranked by publication year and sample size (see Additional file 9A–C).

**Table 2:** Characteristics of the included studies for estimating the discrimination of the Gail model

Reference	Author	Publication year	Geographic background	Study design	Gail model version	5/10-year risk	Sample size	Mean age (years)	Study population	Risk for breast cancer^a^	Time period	Follow-up period	C-statistic/AUC (95% CI)
[11]	Rockhill	2001	America	Cohort	2	5	82,109	45–71	Nurses’ Health Study (NHS)	General population	1992–1997	5.0	0.58 (0.56–0.60)
[30]	Amir	2003	United Kingdom	Cohort	2	10	3150	21–73	Women attending the Family History Screening Programme in University Hospital of South Manchester	Not defined	1987–2001	5.0	0.74 (0.67–0.80)
[21]	Tice	2005	America	Cohort	2	5	81,777	55.9	Community-based registry San Francisco Mammography Registry (SFMR)	General population	1993–2002	5.1 (0.1–15)	0.67 (0.65–0.68)
[29]	Decarli	2006	Italy	Cohort	2	5	10,031	35–64	Florence—European Prospective Investigation Into Cancer and Nutrition cohort (EPIC)	General population	1993–2002	9.0	0.59 (0.55–0.63)
[36]	Crispo	2008	Italy	Case–control	1	5	1765	53.7	National Cancer Institute of Naples (southern Italy)	NA	1997–2000	NA	0.55 (0.53–0.58)
[15]	Tice	2008	America	Cohort	2	5	251,789	40–74	National Cancer Institute-funded Breast Cancer Surveillance Consortium (BCSC)	General population	since 1994	5.3	0.61 (0.60–0.62)
[42]	Pan	2009	China	Cross-sectional	1	5	2133	> 35	Breast cancer risk assessment, evaluation and health education program, in Beijing and Guangzhou community	NA	2006–2007	NA	0.64 (0.61–0.67)
[43]	Liu	2010	China	Cross-sectional	2	5	246	49.82	High-risk breast cancer screening model and chemical intervention study at the community level	NA	2007–2009	NA	0.56 (0.49–0.64)
[44]	Wang	2010	China	Case–control	1	5	228	32–75	Shenzhou Hospital of Shenyang Medical College-based breast cancer cases and control	NA	1998–2007	NA	0.93 (0.89–0.97)
[17]	Tarabishy	2011	America	Cohort	2	5	4726	18–85	Mayo Benign Breast Disease (BBD)	High risk	1982–1991	16.2	0.64 (0.62–0.66)
[22]	Vacek	2011	America	Cohort	1	5	19,779	> 70	Vermont Breast Cancer Surveillance System (VBCSS)	High risk	2001–2009	7.1	0.54 (0.52–0.56)
[27]	Banegas	2012	America	Cohort	2	5	128,976	63.51	Women’s Health Initiative (WHI)	General population	1993–2005	5.0	0.58 (0.57–0.59)
[23]	Quante	2012	America	Cohort	2	10	1857	44	Women with high risk for breast or ovarian cancer in New York site of the Breast Cancer Family Registry (BCFR)	High risk	1995–2011	8.1	0.63 (0.58–0.69)
[32]	Pastor-Barriuso	2013	Spain	Cohort	2	5	54,649	45–68	Population-based Navarre Breast Cancer Screening Program (NBCSP)	General population	1996–2005	7.7	0.54 (0.52–0.57)
[51]	Dite	2013	Australia	Case–control	2	5	1425	45.4	Cases and controls from the Australian Breast Cancer Family Registry (ABCFR)	NA	1992–1998	NA	0.58 (0.55–0.61)
[40]	Anothaisintawee	2013	Thailand	Cross-sectional	NA	NA	15,718	NA	Ramathibodi Hospital and two tertiary hospitals	NA	2011–2013	NA	0.41 (0.36–0.46)
[24]	Ronser	2013	America	Cohort	2	5	11,419	54.0 ± 3.3	Postmenopausal women in California Teachers Study (CTS)	High risk	1995–2009	5.0	0.55 (0.53–0.56)
[41]	Min-1	2014	Korea	Cohort	2	5	40,229	> 10	Breast cancer screening patients routinely screened in Women’s Healthcare Center of Cheil General Hospital	Not defined	1999–2004	5	0.55 (0.50–0.59)
[41]	Min-2	2014	Korea	Cohort	3	5	40,229	> 10	Breast cancer screening patients routinely screened in Women’s Healthcare Center of Cheil General Hospital	Not defined	1999–2004	5	0.54 (0.50–0.59)
[18]	Powell	2014	America	Cohort	2	5	12,843	NA	Marin Women’s Study with high rate of breast cancer, null parity and delayed childbirth	High risk	2003–2007	7.7	0.62 (0.59–0.66)
[45]	Duan	2014	China	Case–control	2	5	400	35–74	Breast cancer cases and controls in the First Affiliate Hospital of KunMing Medical University	NA	2007–2011	NA	0.54 (0.49–0.60)
[19]	McCarthy	2015	America	Cohort	2	5	464	48.7 ± 13	Women referred for biopsy with abnormal (Breast Imaging Reporting And Data System, BI-RADS 4) mammograms at the Hospital of the University of Pennsylvania	High risk	2003–2012	5.0	0.71 (0.65–0.78)
[34]	Dartois-1	2015	France	Cohort	2	5	5843	42–72	Premenopausal Women in French E3N (E´ tude E´ pide´miologique aupre`s des femmes de laMutuelle Ge´ne´rale de l’E´ ducation Nationale (MGEN)) prospective cohort to investigate the cancer risk factors	General population	1993–1998	5.0	0.61 (0.55–0.68)
[34]	Dartois-2	2015	France	Cohort	2	5	7331	42–72	Postmenopausal Women in French E3N (E´ tude E´ pide´miologique aupre`s des femmes de laMutuelle Ge´ne´rale de l’E´ ducation Nationale (MGEN)) prospective cohort to investigate the cancer risk factors	High risk	1993–1998	14.0	0.55 (0.50–0.60)
[39]	Hu	2015	China	Cohort	2	5	42,908	35–69	Women participated in the breast cancer screening in Zhejiang eastern coastal areas of China	General population	2008–2014	5.0	0.59 (0.47–0.70)
[35]	Brentnall	2015	United Kingdom	Cohort	2	10	50,628	47–73	15 screening areas in Greater Manchester, UK	General population	2009–2014	3.2	0.54 (0.52–0.56)
[20]	Schonberg-1	2015	America	Cohort	2	5	71,293	70.0 ± 7.0	Nurses’ Health Study (NHS)	High risk	2004–2009	5.0	0.57 (0.55–0.58)
[20]	Schonberg-2	2015	America	Cohort	2	5	79,611	71.0 ± 6.8	Women’s Health Initiative (WHI), extensive study	High risk	2005–2010	5.0	0.58 (0.56–0.59)
[46]	Rong	2016	China	Case–control	1	5	816	48.9	Breast cancer cases and controls in the Shenzhen Maternal and Child Health Care hospital	NA	2011–2013	NA	0.69 (0.68–0.71)

Note: Gail model type 1, original Gail model; Gail model type 2, modified Gail model for Caucasian-American; Gail model type 3, modified Gail model for Asian-American

*AUC* area under the area under the curve, *CI* confidence interval, *NA* not available

^a^Cohort studies enrolled women with high risk for breast cancer (with higher average age (> 70 years), abnormal breast density, postmenopausal state, breast cancer relatives or high rate of delayed childbirth) were defined as “High risk”; cohort studies that did not accurately depict the characteristics of the participants were defined as “Not defined”. Case–control studies and cross-sectional studies were defined as not available

When stratified by geographic region, the pooled AUCs in American, European and Asian women were 0.60 (95% CI 0.58–0.62), 0.58 (95% CI 0.55–0.60) and 0.61 (95% CI 0.52–0.69), respectively, with no significant heterogeneities (*p* = 0.30) (Fig. 3b–d and see Additional file 10). Subgroup analysis also showed that the pooled AUC in studies with sample size ≥ 10,000 was lower (0.57 vs 0.64; *p* = 0.01). However, the combined AUC was not markedly changed when stratified by other factors (see Additional file 10). The funnel plot indicated no publication bias (Egger’s regression coefficient = −1.25; *p* = 0.54) (see Additional file 11A). According to the trim-and-fill method, eight studies had to be trimmed and the adjusted pooled AUC was 0.63 (95% CI 0.60–0.65) after trimming (see Additional file 11B). In addition, when stratified by geographic region, the funnel plot found significant publication bias across the studies in Europe (Egger’s regression coefficient = 4.45; *p* = 0.01) (see Additional file 12). After trimming, the adjusted AUC in European women was 0.59 (95% CI 0.56–0.62).

Results also showed the pooled AUC for Gail model 1 was 0.70 (95% CI 0.57–0.77), and when stratified by the geographic region the pooled AUCs for Gail model 1 in American and European women [22, 36] and Asian females [42, 44, 46] were 0.55 (95% CI 0.53–0.56) and 0.75 (95% CI 0.63–0.88), respectively (see Additional files 10 and 13). Additionally, the pooled AUC for Gail model 2 was 0.59 (95% CI 0.57–0.61), and when stratified by the geographic region and different versions of Gail model 2 the pooled AUCs for Caucasian-American Gail model 2 in American [15, 17–21, 23, 24, 27], Asian [39, 41, 43, 45] and European [29, 30, 32, 34, 35, 51] females were 0.61 (95% CI 0.59–0.63), 0.55 (95% CI 0.52–0.58) and 0.58 (95% CI 0.55–0.62), respectively (see Additional file 13). However, only one study clearly stated that they used Asian-American Gail model 2, and the AUC was reported to be 0.54 (95% CI 0.50–0.59) [41].

### Diagnostic accuracy of the Gail model

Thirteen studies [11, 15, 25, 26, 31, 33, 37, 44, 45, 47–50] with 783,601 participants were included in this diagnostic meta-analysis (Table 3). The combined sensitivity, specificity and pooled DOR were 0.43 (95% CI 0.24–0.64), 0.88 (95% CI 0.81–0.92), and 5.44 (95% CI 2.17–13.63), respectively (Fig. 3e). Deeks’ funnel plot suggested that publication bias existed among the studies (*p* = 0.026) (see Additional file 14A).

**Table 3:** Characteristics of the included studies for estimating the diagnostic accuracy of the Gail model

Ref	Author	Publication year	Geographic background	Study design	Gail model version	Sample size	Mean age (years)	Study population	Time period	TP	FP	FN	TN
[11]	Rockhill	2001	America	Cohort	2	82,109	45–71	Nurses’ Health Study (NHS)	1992–1997	596	27,457	758	53,298
[25]	Weik	2005	America	Cross-sectional	2	543	20–80	Women who underwent a stereotactic or ultrasound-guided breast biopsy examination	2001–2003	58	95	81	309
[26]	Adams-Campbell-1	2007	America	Nested case–control	1	1450	21–69	Black Women’s Health Study (BWHS)	1995–2003	130	102	595	623
[26]	Adams-Campbell-2	2007	America	Nested case–control	3	1450	21–69	Black Women’s Health Study (BWHS)	1995–2003	30	19	695	706
[31]	Chlebowski	2007	America	Cohort	2	64,568	63	Predicted ER-positive breast cancer in Women’s Health Initiative (WHI) study	1993–2005	462	22,276	461	41,369
[15]	Tice	2008	America	Cohort	2	629,229	40–74	National Cancer Institute-funded Breast Cancer Surveillance Consortium (BCSC)	Since1994	2442	103,852	6342	516,593
[47]	Li	2009	China	Case–control	2	420	40–75	Bao’an Maternal and Child Health Care Hospital, Shenzhen	2003–2008	98	20	5	297
[44]	Wang	2010	China	Case–control	1	228	56	Breast cancer and controls in Shenzhou Hospital of Shenyang Medical College	1998–2007	65	10	4	149
[37]	Ulusoy	2010	Turkey	Case–control	2	1290	49.49	Breast cancer and controls in Ankara University School of Medicine	2002–2008	87	51	563	589
[33]	Buron	2013	Spain	Cohort	2	2200	49–64	Participants with a positive screening mammogram in “Parc de Salut Mar” breast cancer screening program	1996–2010	24	449	28	1161
[48]	Zhou	2014	China	Case–control	1	280	48.62	Breast cancer and controls in Huangpu District in Shanghai of China	2010	72	11	71	126
[45]	Duan	2014	China	Case–control	2	400	52.58	Breast cancer and controls in the First Affiliated Hospital of KunMing Medical University	2007–2011	24	7	176	193
[49]	Huang	2015	China	Case–control	1	317	54.1	Breast cancer and controls in Guangxi Maternal and Child Health Care Hospital	2012–2014	116	13	41	147
[50]	An	2016	China	Case–control	2	567	> 40	Breast cancer and controls in China Japan Union Hospital of Jilin University	2011–2015	207	105	93	162

Note: Gail model type 1, original Gail model; Gail model type 2, modified Gail model for Caucasian-American; Gail model type 3, modified Gail model for African-American

*TP* true positive, *FP* false positive, *FN* false negative, *TN* true negative, *ER* estrogen receptor

When stratified by geographic region, the pooled sensitivity, specificity and DOR in American and European women were 0.26 (95% CI 0.15–0.42), 0.84 (95% CI 0.72–0.91) and 1.79 (95% CI 1.57–2.05), respectively (Fig. 3f) and Deeks’ funnel plot showed no publication bias (*p* = 0.50) (see Additional file 14B). With respect to Asian women, the pooled sensitivity, specificity and DOR were 0.63 (95% CI 0.30–0.87), 0.91 (95% CI 0.84–0.95) and 17.56 (95% CI 4.13–74.59), respectively (Fig. 3g). However, publication bias persisted (*p* = 0.019) (see Additional file 14C).

When further stratified by different versions of the Gail model, the pooled sensitivity, specificity and DOR of Gail model 1 were 0.63 (95% CI 0.27–0.89), 0.91 (95% CI 0.87–0.94) and 17.38 (95% CI 2.66–113.70), respectively, and the corresponding indexes of Gail model 2 were 0.35 (95% CI 0.17–0.59), 0.86 (95% CI 0.76–0.92) and 3.38 (95% CI 1.40–8.17), respectively (see Additional file 15). When subgrouped by different versions of Gail model 2, the pooled sensitivity, specificity and DOR of the Caucasian-American Gail model for American and European women [11, 15, 25, 31, 33] were 0.36 (95% CI 0.27–0.45), 0.77 (95% CI 0.67–0.84) and 1.81 (95% CI 1.66–1.96), respectively, and for Asian females were 0.49 (95% CI 0.11–0.88), 0.90 (95% CI 0.76–0.96) and 8.80 (95% CI 1.19–64.81), respectively [37, 45, 47, 50] (see Additional file 16). However, only one study stated that they used the African-American Gail model and the sensitivity and specificity were reported to be 0.04 (95% CI 0.03–0.05) and 0.97 (95% CI 0.96–0.98), respectively [26]. Subgroup analysis also indicated that the pooled sensitivity with sample size < 1000 was higher than that in studies with ≥ 1000 samples, and the pooled specificity in studies with case–control design, sample size < 1000 and study quality < 8 points was higher than each of their counterparts (see Additional file 17).

### Performance of the Gail model after excluding studies published in Chinese

When excluding studies retrieved in the WANFANG, VIP and CNKI databases, no effect was found on the calibration of Gail model 1. The E/O ratios of the Caucasian-American Gail model and the Asian-American Gail model for Asian women were reported as 2.46 (95% CI 2.10–2.88) and 1.82 (95% CI 1.68–2.04), respectively (see Additional file 18A).

The pooled AUC for Gail model 1 was 0.55 (95% CI 0.53–0.56). After excluding studies published in Chinese, only one study validated discrimination of Asian-American Gail model 2 and Caucasian-American Gail model 2 for Asian females and the AUCs were shown as 0.54 (95% CI 0.50–0.58) and 0.55 (95% CI 0.50–0.60), respectively [41] (see Additional file 18B).

For the diagnostic accuracy of the Gail model, after excluding studies conducted in China, the pooled sensitivity, specificity and the DOR of the Gail model were 0.24 (95% CI 0.14–0.38), 0.85 (95% CI 0.75–0.92) and 1.79 (95% CI 1.58–2.03), respectively. When stratified by different versions of the Gail model, the sensitivity, specificity and the DOR of the Caucasian-American Gail model were 0.25 (95% CI 0.14–0.41), 0.85 (95% CI 0.72–0.93) and 1.89 (95% CI 1.68–2.13), respectively. Only one study remained to evaluate the performance of Gail model 1, and the sensitivity and specificity were reported as 0.15 (95% CI 0.18–0.21) and 0.86 (95% CI 0.83–0.88), respectively [26] (see Additional file 19).

## Discussion

The current study comprehensively evaluated the calibration, discrimination and diagnostic accuracy of different versions of the Gail model. Gail model 1 and Caucasian-American Gail model 2 accurately predicted breast cancer risk for American and European women. However, the Caucasian-American and Asian-American Gail models overpredicted the risk for developing breast cancer about two times in Asian females. TSA showed that evidence in Asian women was sufficient; nonetheless, the results in American and European women need further verification. Moreover, the discrimination and the diagnostic accuracy of any versions of the Gail model were not satisfactory overall or stratified by geographic region.

The current study showed that both the Caucasian-American and the Asian-American Gail models overpredicted the risk for developing breast cancer in Asian women. To explain the results, firstly, the Gail model was constructed based on American white females, but the incidence of breast cancer in Asia (29.1/100,000) was much lower than that in American women (69.9/100,000) [1]. Accordingly, during a specific period, Asian women might not present with so many breast cancer incident cases as expected, leading to a higher E/O ratio. Secondly, the distributions of factors included in the Gail model were different between Asian and American women. Morabia and Costanza [79] conducted an international comparison on reproductive factors in 1998 and found age at first live birth in Asian women was older than that in American females, which may present a higher risk prediction in Asia according to the Gail model [3, 12]. Another potential explanation was the lack of regular breast cancer screening in Asian women. In America, conventional mammography examination would be conducted for women aged 45–74 years every 1 or 2 years [80, 81] and the Gail model was constructed based on women with annual screening [3, 12]. However, routine screening was seldom conducted in Asian women [82]; many of the breast cancer patients could not be detected and resulted in a lower number of observed breast cancer than actually existed, resulting in a higher E/O ratio.

Gail model 1 was designed for white women who were being screened annually [3]. The current version of Gail model 2 used Surveillance Epidemiology and End Results (SEER) breast cancer rates for Asian-American women and the relative and attributable risks were derived from Asian-American females [8]. The Breast Cancer Risk Assessment Tool program specifically warns against the use of the Gail model in Asian women, where breast cancer rates are lower than those in Asian-American women [1]. Accordingly, the risk prediction of the Gail model should be explained with caution when applying it to Asian women and it is necessary to modify the Gail model based on the special risk factors and incidence of breast cancer in Asia, to improve its performance.

For the discrimination of the Gail model, results showed that the pooled AUC was moderately acceptable, while substantial heterogeneities exist between studies. Sample size could partly explain the phenomenon, and two studies with extreme value markedly affected the results. Anothaisintawee et al. [40] reported that the AUC of the Gail model was 0.41 with sample size > 1000, while the study conducted by Wang et al. [44] showed the AUC was 0.93 with < 1000 participants. Subgroup analysis showed no heterogeneities in sample size (≥ 1000 and < 1000) when these two datasets were excluded (0.62 vs 0.58; *p* = 0.07).

Previous meta-analyses also showed similar results that the Gail model had a satisfactory calibration and moderately acceptable discrimination [53–55]. Besides, the current study evaluated the diagnostic accuracy of the Gail model and the results showed that the sensitivity of the Gail model was poor and the results were even worse when focusing on the studies in American and European women. Accordingly, many of the breast cancer cases were misdiagnosed and this may partly explain the modest discrimination of the Gail model to some extent. Other risk factors for breast cancer such as mammographic density [83] and genetic factors [84] should be added to the Gail model in the future to provide a more accurate prediction of breast cancer. Nonetheless, few studies were combined to evaluate the diagnostic accuracy of the Gail model; more related studies are required to further confirm the results in the future.

Diagnostic meta-analysis also showed that the pooled specificity was higher in Asian women than that in American and European women, and studies with a case–control design, sample size < 1000 and study quality < 8 points presented a higher specificity than each of their counterparts. All studies in Asia were conducted using the hospital-based case–control design and the healthy controls were prone to have fewer risk factors than the cases. For example, biopsy is required for breast cancer cases, but is rarely used in healthy women in Asia; this may lead to lower prediction of risk in controls according to the Gail model and may increase the true negative rate and the specificity value. Moreover, most of the case–control studies were conducted with smaller sample sizes and lower study quality, and thus the difference in these subgroups may be partly explained by the distorted distribution of the case–control studies.

Additionally, Deeks’ funnel plot showed publication bias exists in Chinese studies, some studies with small sample size and lower DOR may not be published, and the diagnostic accuracy of the Gail model may be overestimated to some extent. Sensitivity analysis showed that when excluding studies conducted in Chinese, the pooled specificity of the Gail model was not significantly altered but the pooled sensitivity and DOR were markedly decreased.

### Limitations

The current study detected substantial heterogeneities across the studies for the three statistics that we summarized; these heterogeneities can be partially explained, but could not be markedly diminished by different geographic regions and various versions of the Gail model. Secondly, although many studies tried to evaluate the performance of different versions of the Gail model, they could not be included in this meta-analysis as they did not provide necessary indexes of the E/O ratio or the AUC with 95% CIs [85, 86]. This limits the power of this meta-analysis to evaluate the performance of different versions of the Gail model. Thirdly, most of the included studies did not clarify which version of Gail model 2 was utilized in their studies. In the current meta-analysis, the American and European studies who cited Constantino et al.’s paper [12] and the Asian studies which were published before the Asian-American Gail model was developed [8] were all deemed to be Caucasian-American Gail model 2. This may lead to misclassification to some extent and may partly affect the precision of the results. Finally, in order to comprehensively evaluate the performance of the Gail model in China, the WANFANG, VIP and CNKI databases were searched, which may partly overestimate the diagnostic accuracy of the Gail model, although it has no significant effect on the Gail model’s calibration and discrimination.

## Conclusions

Although the original Gail model 1 and the Caucasian-American Gail model had a well-fitting calibration in American and European women, the Caucasian-American and Asian-American Gail models may overestimate the risk in Asian females about two times. Moreover, the discrimination and diagnostic accuracy of the Gail model were not satisfactory overall or stratified by geographic region and different versions of the Gail model. Accordingly, the Gail model was appropriate for predicting the incidence of breast cancer in American and European women, but not suitable for use in Asian women. Furthermore, this model cannot tell a woman whether she will or will not develop breast cancer precisely. Even so, it is still very valuable for women to have a well-calibrated risk assessment and select different prevention strategies that are suitable for their risk level.

## Additional files

Additional file 1: Shows sensitivity analysis (A), cumulative meta-analysis ranked by publication year (B) and sample size (C) of the calibration of the Gail model. (PDF 1593 kb)

Additional file 2: Shows funnel plot of calibration of the Gail model (shows) and funnel plot adjusted by trim-and-fill method (B). (PDF 255 kb)

Additional file 3: Shows subgroup analysis of calibration of the Gail model. (PDF 103 kb)

Additional file 4: Shows funnel plot of calibration of the Gail model when stratified by geographic region in America (A), Europe (B) and Asia (C). (PDF 305 kb)

Additional file 5: Shows forest plot of calibration of the Asian-American version of Gail model 2 in Asian females and Caucasian-American Gail model 2 in American, Asian and European women. (PDF 663 kb)

Additional file 6: Shows forest plot (A), sensitivity analysis (B) and cumulative analysis ranked by publication year (C) and sample size (D) of calibration of the Gail model after excluding studies conducted in Asian women. (PDF 1202 kb)

Additional file 7: Shows pooled E/O ratio for Gail model 1 and Caucasian-American Gail model 2 after excluding studies conducted in Asian women. (PDF 582 kb)

Additional file 8: Shows subgroup analysis of calibration of the Gail model after excluding studies conducted in Asian women. (PDF 106 kb)

Additional file 9: Shows sensitivity analysis (A), cumulative meta-analysis ranked by publication year (B) and sample size (C) of discrimination of the Gail model. (PDF 1321 kb)

Additional file 10: Shows subgroup analysis of discrimination of the Gail model. (PDF 104 kb)

Additional file 11: Shows funnel plot of discrimination of the Gail model (**A**) and funnel plot adjusted by trim-and-fill method (B). (PDF 226 kb)

Additional file 12: Shows funnel plot of discrimination of the Gail model when stratified by geographic region in America (A), Europe and others (B) and Asia (C). (PDF 278 kb)

Additional file 13: Shows pooled AUC for Caucasian-American Gail model 2 in American, Asian and European women and Gail model 1 in American and European women and Asian females. (PDF 686 kb)

Additional file 14: Shows Deeks’ funnel plot of diagnostic accuracy meta-analysis (A) and funnel plot of stratified analysis in America and Europe (B) and Asia (C). (PDF 294 kb)

Additional file 15: Shows pooled sensitivity, specificity and DOR of Gail model 1 (A) and Gail model 2 (B). (PDF 994 kb)

Additional file 16: Shows pooled sensitivity, specificity and DOR of the Caucasian-American Gail model in American and European women (A) and Asian females (B). (PDF 828 kb)

Additional file 17: Shows subgroup analysis of diagnostic accuracy of the Gail model. (PDF 376 kb)

Additional file 18: Shows calibration (A) and discrimination (B) of different versions of the Gail model after excluding studies published in Chinese. (PDF 531 kb)

Additional file 19: Shows pooled sensitivity, specificity and DOR of Gail model 1 and Caucasian-American Gail model 2 after excluding studies published in Chinese. (PDF 888 kb)

## Abbreviations

AUC: Area under the curve
BCRAT: Breast Cancer Risk Assessment Tool
CI: Confidence interval
CNKI: China National Knowledge Infrastructure
C-statistic: Concordance statistic
DOR: Diagnostic odds ratio
E/O: Expected-to-observed ratio
FN: False negative
FP: False positive
MeSH: Medical subject headings
NOS: Newcastle–Ottawa Scale
QUADAS: Quality Assessment Diagnostic Accuracy Studies
RIS: Required information size
SEER: Surveillance Epidemiology and End Results
TN: True negative
TP: True positive
TSA: Trial sequential analysis
