# Supplementary material for: Assessment of performance of the Gail model for predicting breast cancer risk: a systematic review and meta-analysis with trial sequential analysis
Source: Breast Cancer Res. 2018 Mar 13;20:18. doi: 10.1186/s13058-018-0947-5 (PMC5850919; doi:10.1186/s13058-018-0947-5)
Supplement: Supplementary file 1 — Shows sensitivity analysis (A), cumulative meta-analysis ranked by publication year (B) and sample size (C) of the calibration of the Gail model. (PDF 1593 kb) [file 13058_2018_947_MOESM1_ESM.pdf]

A

Study name	E/O ratio	Lower limit	Upper limit	Z-Value	p-Value
	Statistics with study removed				
Bondy 1994	1.16	1.04	1.29	2.69	0.01
Spiegelman 1994	1.16	1.04	1.29	2.70	0.01
Costantino 1999-1	1.18	1.06	1.32	2.95	0.00
Costantino 1999-2	1.17	1.05	1.31	2.81	0.00
Rockhill 2001	1.17	1.05	1.31	2.82	0.00
Amir 2003	1.18	1.06	1.32	3.05	0.00
Bernatsky 2004	1.19	1.06	1.32	3.10	0.00
Olson 2004	1.16	1.04	1.29	2.68	0.01
Boyle 2004	1.16	1.04	1.30	2.75	0.01
Decarli 2006	1.17	1.05	1.31	2.88	0.00
Chlebowski 2007	1.18	1.05	1.33	2.87	0.00
Tice 2008	1.18	1.04	1.33	2.63	0.01
Schonfeld 2010-1	1.18	1.04	1.33	2.66	0.01
Schonfeld 2010-2	1.18	1.05	1.32	2.87	0.00
Tarabishy 2011	1.17	1.05	1.31	2.88	0.00
Chay 2012-1	1.13	1.02	1.26	2.30	0.02
Chay 2012-2	1.14	1.03	1.27	2.49	0.01
MacInnis 2012	1.18	1.05	1.31	2.91	0.00
Pastor-Barriuso 2013	1.15	1.04	1.29	2.62	0.01
Buron 2013	1.20	1.08	1.33	3.32	0.00
Min 2014-1	1.13	1.02	1.26	2.31	0.02
Min 2014-2	1.16	1.04	1.29	2.67	0.01
Powell 2014	1.18	1.06	1.32	2.98	0.00
McCarthy 2015	1.12	1.01	1.25	2.15	0.03
Dartois 2015	1.17	1.05	1.31	2.85	0.00
Hu 2015	1.14	1.02	1.27	2.40	0.02
Brentnall 2015	1.13	1.02	1.24	2.44	0.01
Schonberg 2015-1	1.16	1.04	1.30	2.70	0.01
Schonberg 2015-2	1.17	1.05	1.31	2.74	0.01
	1.16	1.05	1.30	2.79	0.01

E/O ratio (95% CI) with study removed

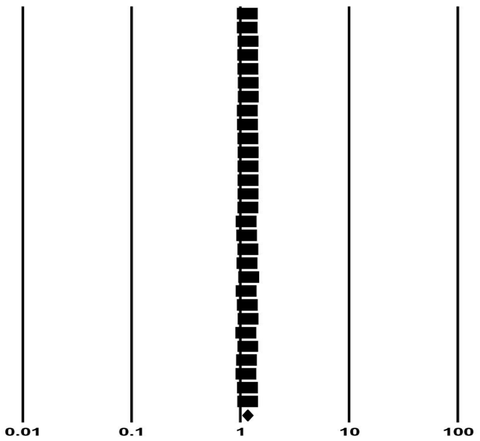

Meta Analysis

B

Study name	Publication year	E/O ratio	Lower limit	Upper limit	Z-Value	p-Value
		Cumulative statistics				
Bondy 1994	1994	1.31	0.96	1.79	1.70	0.09
Spiegelman 1994	1994	1.33	1.28	1.38	14.96	0.00
Costantino 1999-1	1999	1.13	0.80	1.59	0.69	0.49
Costantino 1999-2	1999	1.10	0.85	1.42	0.74	0.46
Rockhill 2001	2001	1.06	0.86	1.32	0.56	0.58
Amir 2003	2003	1.00	0.81	1.23	-0.02	0.99
Bernatsky 2004	2004	0.94	0.77	1.15	-0.61	0.54
Olson 2004	2004	0.97	0.80	1.18	-0.28	0.78
Boyle 2004	2004	0.99	0.83	1.19	-0.08	0.93
Decarli 2006	2006	0.99	0.84	1.16	-0.17	0.86
Chlebowski 2007	2007	0.96	0.80	1.15	-0.45	0.65
Tice 2008	2008	0.95	0.84	1.09	-0.71	0.48
Schonfeld 2010-1	2010	0.95	0.85	1.05	-1.04	0.30
Schonfeld 2010-2	2010	0.94	0.85	1.03	-1.32	0.19
Tarabishy 2011	2011	0.94	0.85	1.03	-1.37	0.17
Chay 2012-1	2012	1.00	0.91	1.10	-0.01	0.99
Chay 2012-2	2012	1.04	0.94	1.16	0.76	0.44
MacInnis 2012	2012	1.03	0.93	1.14	0.63	0.53
Pastor-Barriuso 2013	2013	1.05	0.95	1.17	0.99	0.32
Buron 2013	2013	1.02	0.92	1.13	0.34	0.73
Min 2014-1	2014	1.06	0.95	1.19	1.10	0.27
Min 2014-2	2014	1.07	0.96	1.19	1.29	0.20
Powell 2014	2014	1.06	0.95	1.18	1.09	0.28
McCarthy 2015	2015	1.11	1.00	1.23	1.88	0.06
Dartois 2015	2015	1.10	0.99	1.22	1.82	0.07
Hu 2015	2015	1.13	1.02	1.25	2.30	0.02
Brentnall 2015	2015	1.17	1.04	1.31	2.64	0.01
Schonberg 2015-1	2015	1.17	1.05	1.31	2.74	0.01
Schonberg 2015-2	2015	1.16	1.05	1.30	2.79	0.01
		1.16	1.05	1.30	2.79	0.01

Cumulative E/O ratio (95% CI)

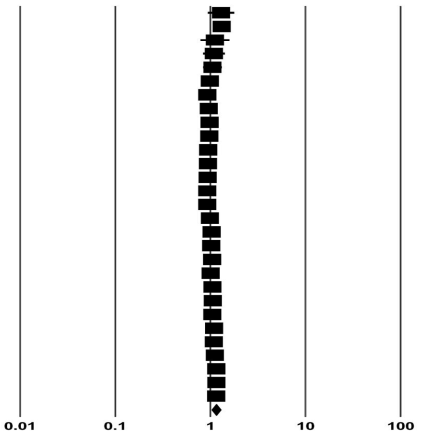

Meta Analysis

C

Study name	Sample size	E/O ratio	Lower limit	Upper limit	Z-Value	p-Value
		Cumulative statistics				
McCarthy 2015	464	3.78	2.78	5.13	8.51	0.00
Olson 2004	674	2.29	0.85	6.20	1.64	0.10
Bernatsky 2004	871	1.37	0.44	4.32	0.54	0.59
Bondy 1994	1981	1.37	0.63	2.98	0.79	0.43
MacInnis 2012	2000	1.25	0.68	2.32	0.72	0.47
Buron 2013	2200	1.09	0.62	1.92	0.31	0.75
Amir 2003	3150	1.02	0.64	1.63	0.10	0.92
Tarabishy 2011	4726	1.01	0.68	1.50	0.05	0.96
Boyle 2004	5383	1.03	0.71	1.48	0.14	0.89
Costantino 1999-1	5969	1.00	0.74	1.36	0.03	0.98
Costantino 1999-2	5969	1.01	0.77	1.32	0.05	0.96
Decarli 2006	10031	1.00	0.79	1.27	-0.01	0.99
Powell 2014	12843	0.98	0.79	1.22	-0.17	0.86
Dartois 2015	13174	0.98	0.80	1.19	-0.20	0.84
Chay 2012-1	28104	1.05	0.82	1.33	0.38	0.70
Chay 2012-2	28104	1.09	0.84	1.41	0.64	0.52
Min 2014-1	40229	1.14	0.88	1.49	0.98	0.32
Min 2014-2	40229	1.15	0.90	1.48	1.10	0.27
Hu 2015	42908	1.19	0.93	1.52	1.37	0.17
Brentnall 2015	50628	1.24	0.94	1.62	1.55	0.12
Pastor-Barriuso 2013	54649	1.25	0.98	1.59	1.80	0.07
Schonfeld 2010-2	64868	1.23	0.99	1.53	1.84	0.07
Schonberg 2015-1	71293	1.23	1.01	1.49	2.05	0.04
Schonberg 2015-2	79611	1.22	1.03	1.45	2.24	0.02
Rockhill 2001	82109	1.21	1.03	1.42	2.30	0.02
Spiegelman 1994	115172	1.21	1.05	1.40	2.61	0.01
Chlebowski 2007	147916	1.19	1.04	1.37	2.46	0.01
Schonfeld 2010-1	181979	1.18	1.04	1.33	2.63	0.01
Tice 2008	629229	1.16	1.05	1.30	2.79	0.01
		1.16	1.05	1.30	2.79	0.01

Cumulative E/O ratio (95% CI)

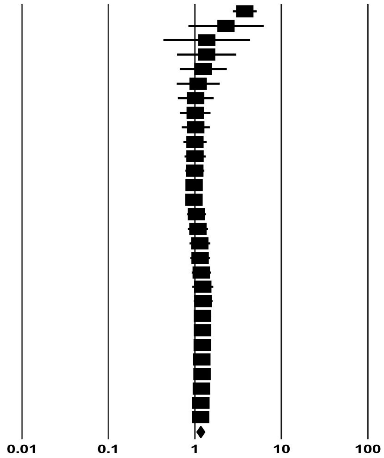

Meta Analysis

Additional file 1. The sensitivity analysis (A), cumulative meta-analysis ranked by the publication year (B) and sample size (C) of the calibration of the Gail model.
