# Supplementary figures and images for: Assessment of performance of the Gail model for predicting breast cancer risk: a systematic review and meta-analysis with trial sequential analysis
Source: Breast Cancer Res. 2018 Mar 13;20:18. doi: 10.1186/s13058-018-0947-5 (PMC5850919; doi:10.1186/s13058-018-0947-5)

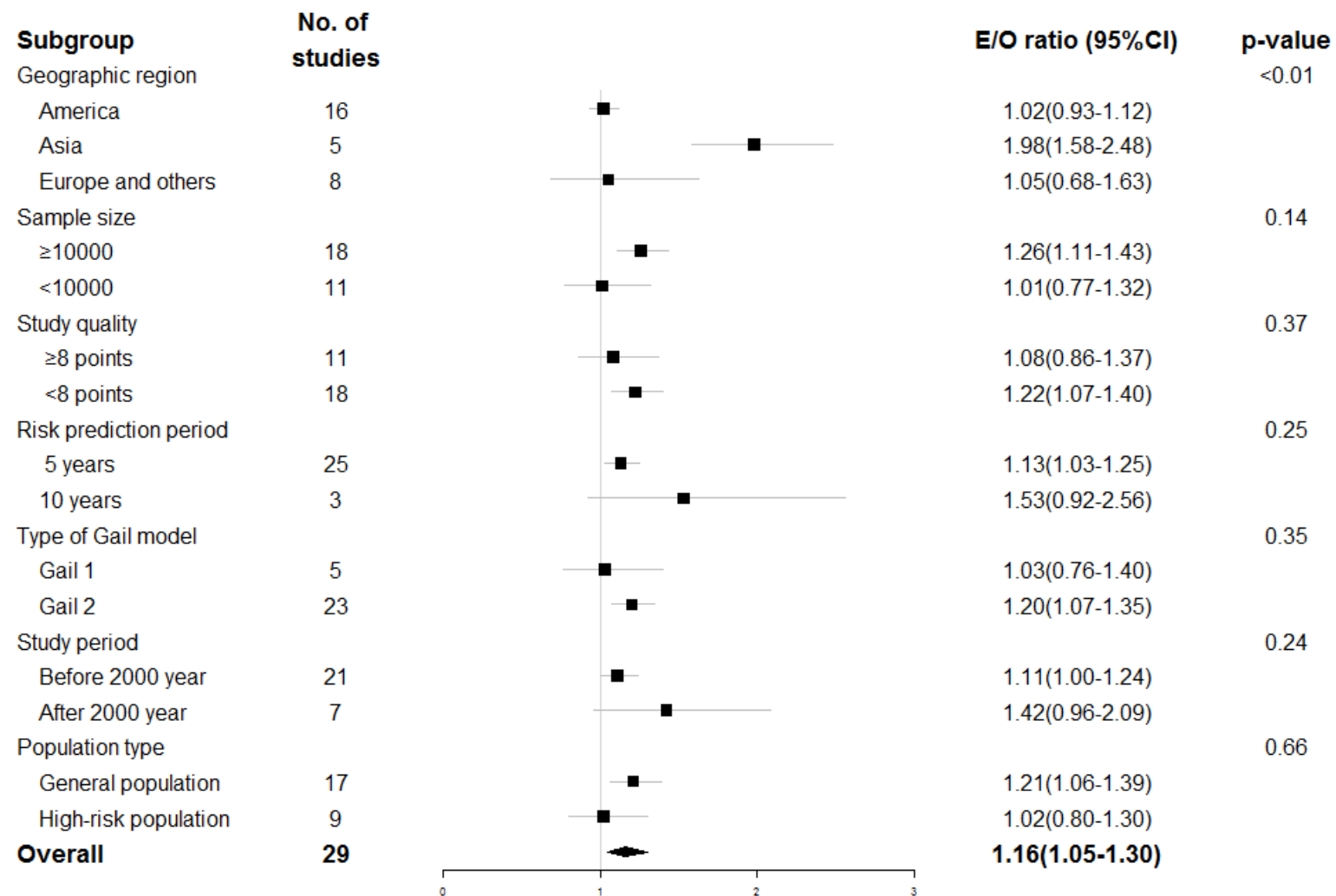

**Additional file 3.** Subgroup analysis of the calibration of the Gail model.

Supplement: Supplementary file 3 — Shows subgroup analysis of calibration of the Gail model. (PDF 103 kb) [file 13058_2018_947_MOESM3_ESM.pdf]

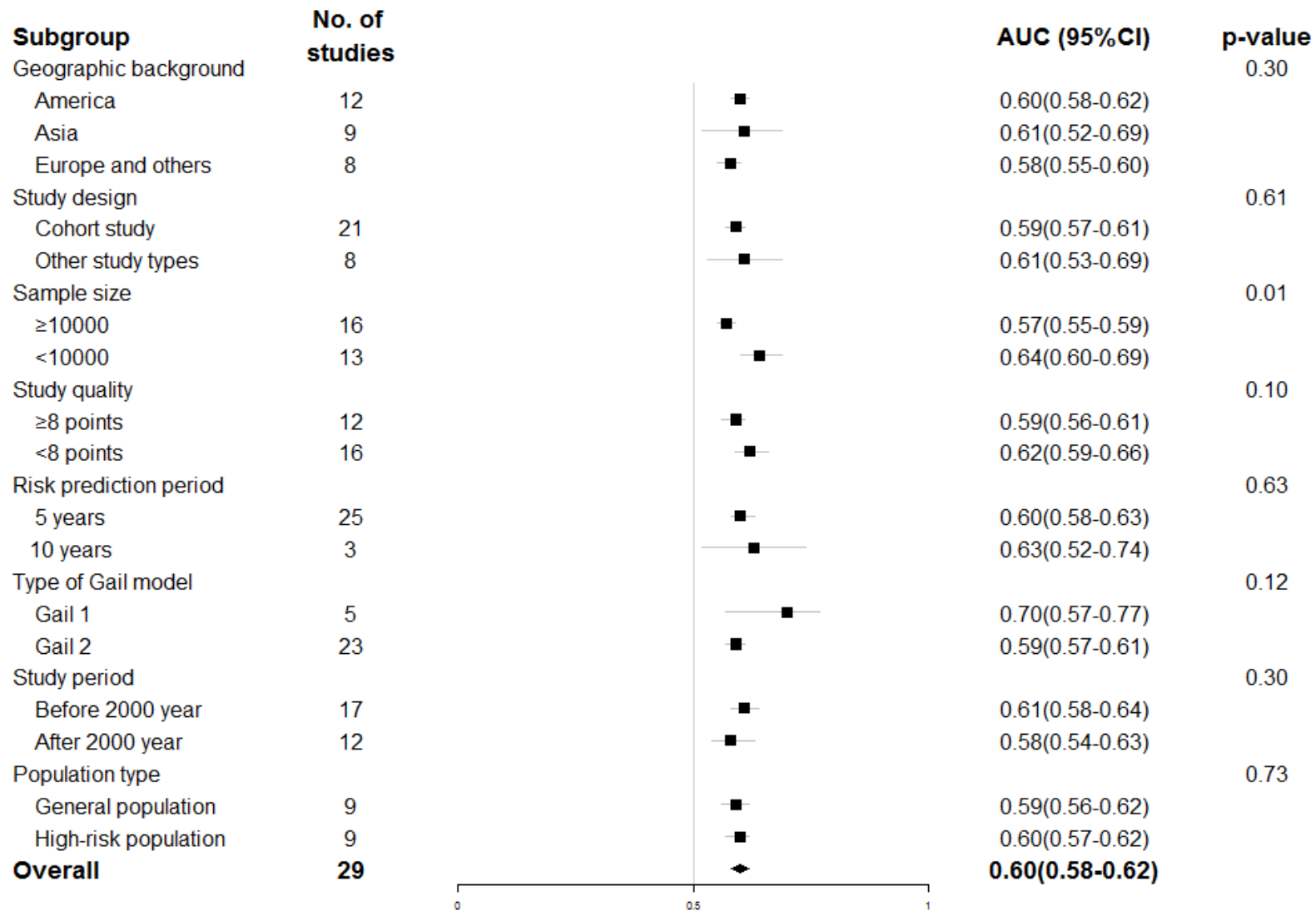

**Additional file 10.** Subgroup analysis of the discrimination of the Gail model.

Supplement: Supplementary file 10 — Shows subgroup analysis of discrimination of the Gail model. (PDF 104 kb) [file 13058_2018_947_MOESM10_ESM.pdf]

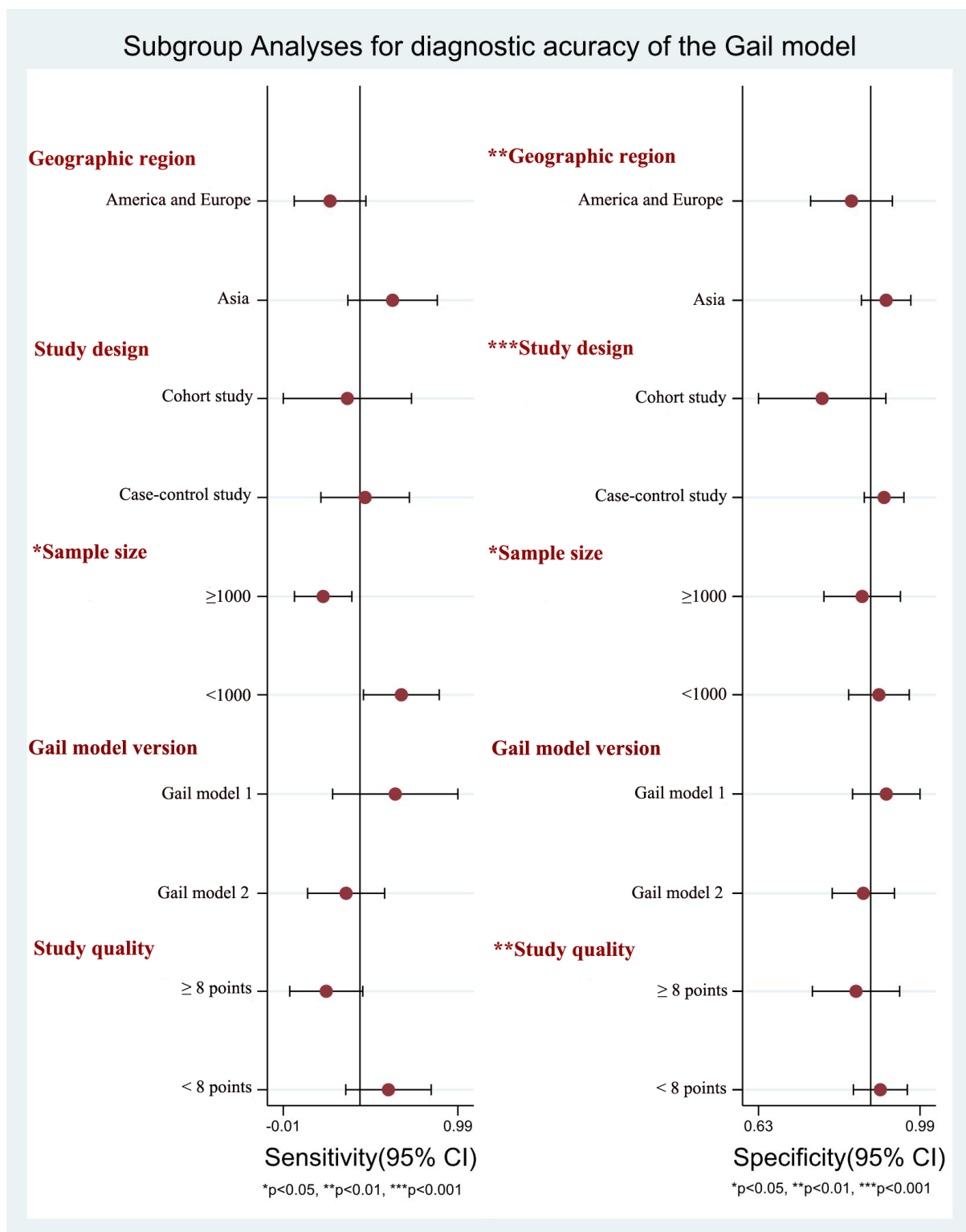

**Additional file 17.** Subgroup analysis of the diagnostic accuracy of the Gail model.

Supplement: Supplementary file 17 — Shows subgroup analysis of diagnostic accuracy of the Gail model. (PDF 376 kb) [file 13058_2018_947_MOESM17_ESM.pdf]
