# Supplementary material for: Assessment of performance of the Gail model for predicting breast cancer risk: a systematic review and meta-analysis with trial sequential analysis
Source: Breast Cancer Res. 2018 Mar 13;20:18. doi: 10.1186/s13058-018-0947-5 (PMC5850919; doi:10.1186/s13058-018-0947-5)
Supplement: Supplementary file 4 — Shows funnel plot of calibration of the Gail model when stratified by geographic region in America (A), Europe (B) and Asia (C). (PDF 305 kb) [file 13058_2018_947_MOESM4_ESM.pdf]

A

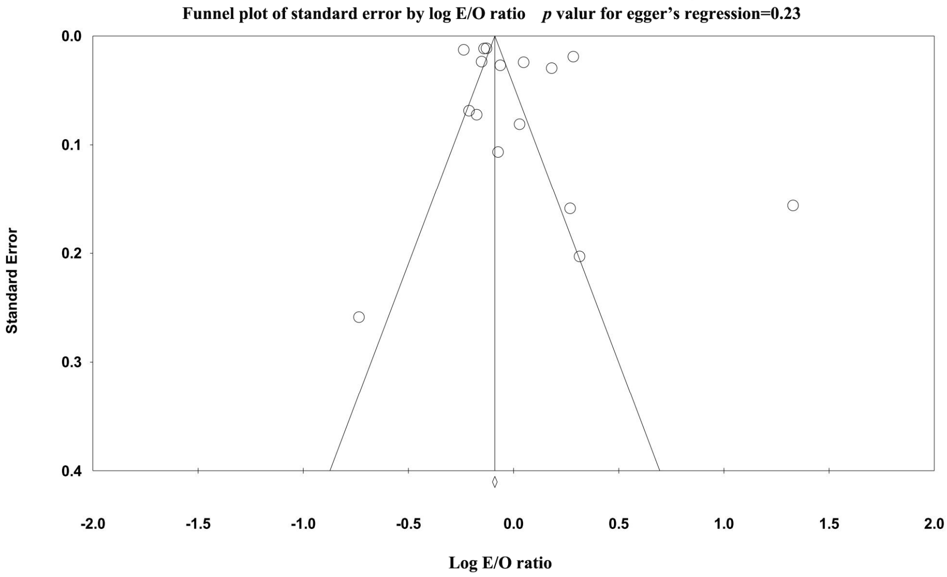

B

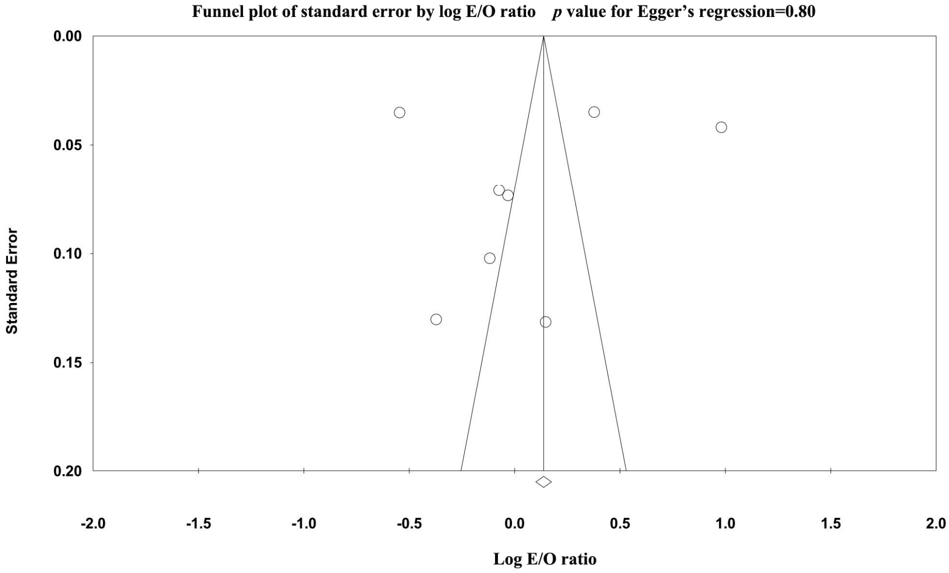

C

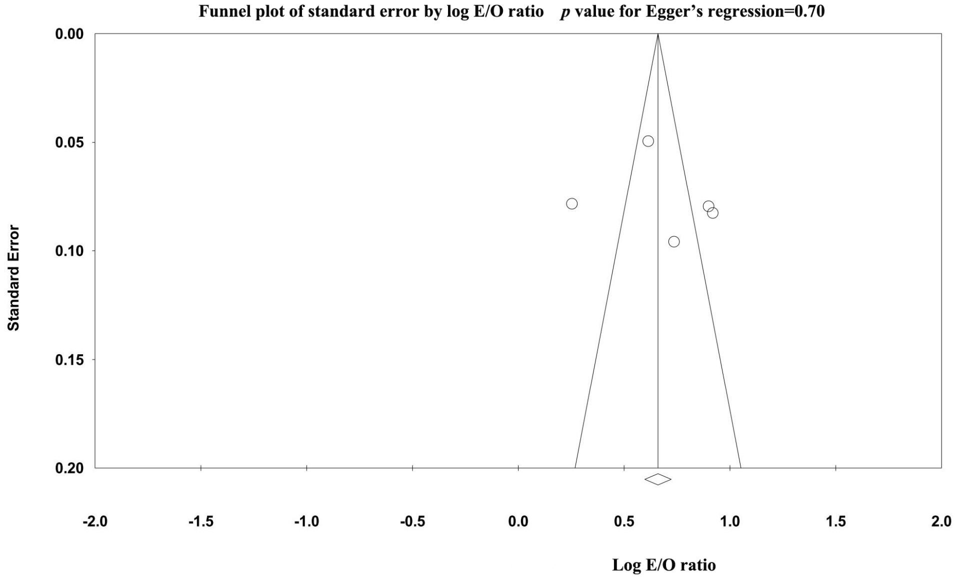

Additional file 4. Funnel plot of the calibration of the Gail model when stratified by geographic region in America (A), Europe and others (B) and Asia (C).
