# Supplementary material for: Assessment of performance of the Gail model for predicting breast cancer risk: a systematic review and meta-analysis with trial sequential analysis
Source: Breast Cancer Res. 2018 Mar 13;20:18. doi: 10.1186/s13058-018-0947-5 (PMC5850919; doi:10.1186/s13058-018-0947-5)
Supplement: Supplementary file 5 — Shows forest plot of calibration of the Asian-American version of Gail model 2 in Asian females and Caucasian-American Gail model 2 in American, Asian and European women. (PDF 663 kb) [file 13058_2018_947_MOESM5_ESM.pdf]

Meta Analysis

Study name	Subgroup within study	E/O ratio	Lower limit	Upper limit	Z-Value	p-Value
Min 2014-2	Asian-American model for Asian	1.29	1.11	1.50	3.24	0.00
Chay 2012-1	Asian-American model for Asian	2.51	2.13	2.95	11.12	0.00
Chay 2012-2	Asian-American model for Asian	1.85	1.68	2.04	12.42	0.00
	**Combined**	1.82	1.31	2.51	3.59	0.00
Costantino 1999-2	Caucasian-American model for American	1.03	0.88	1.21	0.36	0.72
Rockhill 2001	Caucasian-American model for American	0.94	0.89	0.99	-2.28	0.02
Chlebowski 2007	Caucasian-American model for American	0.79	0.77	0.81	-18.25	0.00
Tice 2008	Caucasian-American model for American	0.88	0.86	0.90	-11.02	0.00
Schonfeld 2010-1	Caucasian-American model for American	0.87	0.85	0.89	-11.87	0.00
Schonfeld 2010-2	Caucasian-American model for American	0.86	0.82	0.90	-6.35	0.00
Tarabishy 2011	Caucasian-American model for American	0.93	0.75	1.15	-0.68	0.50
Powell 2014	Caucasian-American model for American	0.81	0.71	0.93	-3.06	0.00
McCarthy 2015	Caucasian-American model for American	3.78	2.78	5.13	8.51	0.00
Schonberg 2015-1	Caucasian-American model for American	1.20	1.13	1.27	6.12	0.00
Schonberg 2015-2	Caucasian-American model for American	1.05	1.00	1.10	2.01	0.04
	**Combined**	0.98	0.91	1.06	-0.43	0.67
Min 2014-1	Caucasian-American model for Asian	2.46	2.10	2.88	11.30	0.00
Hu 2015	Caucasian-American model for Asian	2.09	1.73	2.52	7.68	0.00
	**Combined**	2.29	1.95	2.68	10.21	0.00
Amir 2003	Caucasian-American model for European	0.69	0.53	0.89	-2.85	0.00
Dartois 2015	Caucasian-American model for European	0.97	0.84	1.12	-0.42	0.68
Boyle 2004	Caucasian-American model for European	1.16	0.90	1.50	1.13	0.26
Decarli 2006	Caucasian-American model for European	0.93	0.81	1.07	-1.02	0.31
Pastor-Barriuso 2013	Caucasian-American model for European	1.46	1.36	1.56	10.81	0.00
Buron 2013	Caucasian-American model for European	0.58	0.54	0.62	-15.46	0.00
Brentnall 2015	Caucasian-American model for European	2.67	2.46	2.90	23.40	0.00
	**Combined**	1.07	0.66	1.74	0.29	0.77
	**Overall combined**	1.18	1.11	1.27	4.82	0.00

Statistics for each study

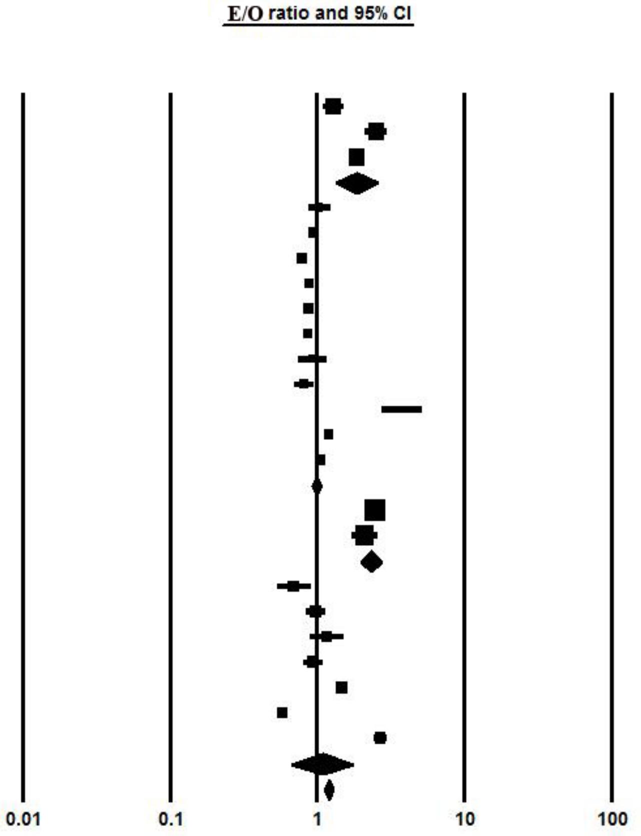

Meta Analysis

Additional file 5. Forest plot of the calibration of the Asian-American version of the Gail model 2 in Asian females and the Caucasian-American Gail model 2 in American, Asian and European women.
