# Supplementary material for: Assessment of performance of the Gail model for predicting breast cancer risk: a systematic review and meta-analysis with trial sequential analysis
Source: Breast Cancer Res. 2018 Mar 13;20:18. doi: 10.1186/s13058-018-0947-5 (PMC5850919; doi:10.1186/s13058-018-0947-5)
Supplement: Supplementary file 6 — Shows forest plot (A), sensitivity analysis (B) and cumulative analysis ranked by publication year (C) and sample size (D) of calibration of the Gail model after excluding studies conducted in Asian women. (PDF 1202 kb) [file 13058_2018_947_MOESM6_ESM.pdf]

A

Meta Analysis

Study name	E/O ratio	Lower limit	Upper limit	Z-Value	p-Value
Bondy 1994	1.31	0.96	1.79	1.70	0.09
Spiegelman 1994	1.33	1.28	1.38	14.86	0.00
Costantino 1999-1	0.84	0.73	0.97	-2.40	0.02
Costantino 1999-2	1.03	0.88	1.21	0.36	0.72
Rockhill 2001	0.94	0.89	0.99	-2.28	0.02
Amir 2003	0.69	0.53	0.89	-2.85	0.00
Olson 2004	1.37	0.92	2.04	1.55	0.12
Bernatsky 2004	0.48	0.29	0.80	-2.84	0.00
Boyle 2004	1.16	0.90	1.50	1.13	0.26
Decarli 2006	0.93	0.81	1.07	-1.02	0.31
Chlebowski 2007	0.79	0.77	0.81	-18.25	0.00
Tice 2008	0.88	0.86	0.90	-11.02	0.00
Schonfeld 2010-2	0.86	0.82	0.90	-6.35	0.00
Schonfeld 2010-1	0.87	0.85	0.89	-11.87	0.00
Tarabishy 2011	0.93	0.75	1.15	-0.68	0.50
MacInnis 2012	0.89	0.73	1.09	-1.14	0.25
Buron 2013	0.58	0.54	0.62	-15.46	0.00
Pastor-Barriuso 2013	1.46	1.36	1.56	10.81	0.00
Powell 2014	0.81	0.71	0.93	-3.06	0.00
McCarthy 2015	3.78	2.78	5.13	8.51	0.00
Dartois 2015	0.97	0.84	1.12	-0.42	0.68
Brentnall 2015	2.67	2.46	2.90	23.40	0.00
Schonberg 2015-1	1.20	1.13	1.27	6.12	0.00
Schonberg 2015-2	1.05	1.00	1.10	2.01	0.04
	1.04	0.93	1.16	0.71	0.48

E/O ratio and 95% CI

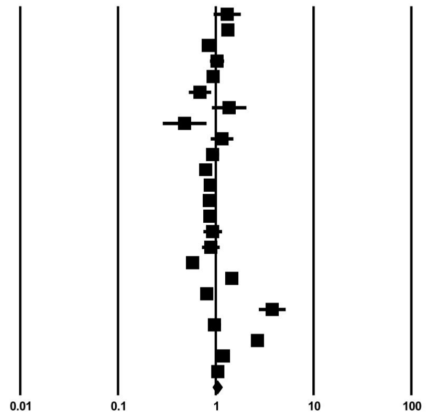

B

Meta Analysis

Study name	E/O	Lower limit	Upper limit	Z-Value	p-Value
Bondy 1994	1.03	0.93	1.15	0.55	0.58
Spiegelman 1994	1.03	0.93	1.14	0.49	0.63
Costantino 1999-1	1.05	0.94	1.17	0.86	0.39
Costantino 1999-2	1.04	0.93	1.16	0.70	0.49
Rockhill 2001	1.04	0.93	1.17	0.76	0.45
Amir 2003	1.06	0.95	1.18	0.98	0.33
Olson 2004	1.03	0.93	1.15	0.55	0.58
Bernatsky 2004	1.06	0.95	1.18	1.03	0.30
Boyle 2004	1.03	0.93	1.15	0.62	0.54
Decarli 2006	1.04	0.94	1.16	0.78	0.44
Chlebowski 2007	1.05	0.94	1.18	0.89	0.37
Tice 2008	1.05	0.93	1.18	0.76	0.45
Schonfeld 2010-2	1.05	0.94	1.17	0.84	0.40
Schonfeld 2010-1	1.05	0.93	1.18	0.77	0.44
Tarabishy 2011	1.04	0.94	1.16	0.78	0.44
MacInnis 2012	1.05	0.94	1.17	0.81	0.42
Buron 2013	1.07	0.96	1.19	1.24	0.21
Pastor-Barriuso 2013	1.02	0.92	1.13	0.40	0.69
Powell 2014	1.05	0.94	1.17	0.90	0.37
McCarthy 2015	0.99	0.89	1.10	-0.12	0.90
Dartois 2015	1.04	0.93	1.16	0.75	0.46
Brentnall 2015	0.99	0.90	1.08	-0.23	0.82
Schonberg 2015-1	1.03	0.93	1.15	0.56	0.57
Schonberg 2015-2	1.04	0.93	1.16	0.67	0.50
	1.04	0.93	1.16	0.71	0.48

Statistics with study removed

E/O ratio (95% CI) with study removed

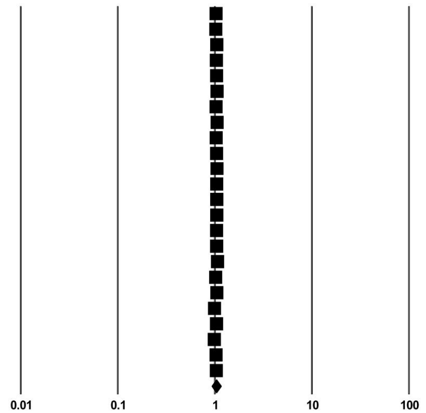

C

Meta Analysis

Study name	E/O	Lower limit	Upper limit	Z-Value	p-Value
Bondy 1994	1.31	0.96	1.79	1.70	0.09
Spiegelman 1994	1.33	1.28	1.38	14.96	0.00
Costantino 1999-1	1.13	0.80	1.59	0.69	0.49
Costantino 1999-2	1.10	0.85	1.42	0.74	0.46
Rockhill 2001	1.06	0.86	1.32	0.56	0.58
Amir 2003	1.00	0.81	1.23	-0.02	0.99
Olson 2004	1.03	0.85	1.25	0.30	0.76
Bernatsky 2004	0.97	0.80	1.18	-0.28	0.78
Boyle 2004	0.99	0.83	1.19	-0.08	0.93
Decarli 2006	0.99	0.84	1.16	-0.17	0.86
Chlebowski 2007	0.96	0.80	1.15	-0.45	0.65
Tice 2008	0.95	0.84	1.09	-0.71	0.48
Schonfeld 2010-2	0.94	0.84	1.06	-0.95	0.34
Schonfeld 2010-1	0.94	0.85	1.03	-1.32	0.19
Tarabishy 2011	0.94	0.85	1.03	-1.37	0.17
MacInnis 2012	0.93	0.85	1.02	-1.49	0.14
Buron 2013	0.90	0.82	0.99	-2.13	0.03
Pastor-Barriuso 2013	0.93	0.85	1.03	-1.38	0.17
Powell 2014	0.93	0.84	1.02	-1.59	0.11
McCarthy 2015	0.98	0.89	1.08	-0.49	0.62
Dartois 2015	0.98	0.89	1.07	-0.51	0.61
Brentnall 2015	1.03	0.92	1.16	0.52	0.60
Schonberg 2015-1	1.04	0.93	1.16	0.67	0.50
Schonberg 2015-2	1.04	0.93	1.16	0.71	0.48
	1.04	0.93	1.16	0.71	0.48

Cumulative statistics

Cumulative E/O ratio (95% CI)

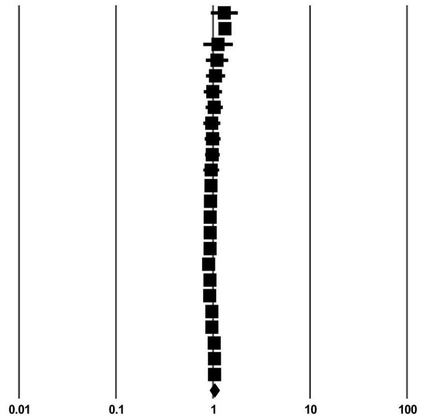

D

Meta Analysis

Study name	E/O	Lower limit	Upper limit	Z-Value	p-Value
McCarthy 2015	3.78	2.78	5.13	8.51	0.00
Olson 2004	2.29	0.85	6.20	1.64	0.10
Bernatsky 2004	1.37	0.44	4.32	0.54	0.59
Bondy 1994	1.37	0.63	2.98	0.79	0.43
MacInnis 2012	1.25	0.68	2.32	0.72	0.47
Buron 2013	1.09	0.62	1.92	0.31	0.75
Amir 2003	1.02	0.64	1.63	0.10	0.92
Tarabishy 2011	1.01	0.68	1.50	0.05	0.96
Boyle 2004	1.03	0.71	1.48	0.14	0.89
Costantino 1999-1	1.00	0.74	1.36	0.03	0.98
Costantino 1999-2	1.01	0.77	1.32	0.05	0.96
Decarli 2006	1.00	0.79	1.27	-0.01	0.99
Powell 2014	0.98	0.79	1.22	-0.17	0.86
Dartois 2015	0.98	0.80	1.19	-0.20	0.84
Brentnall 2015	1.05	0.76	1.45	0.32	0.75
Pastor-Barriuso 2013	1.08	0.81	1.43	0.51	0.61
Schonfeld 2010-2	1.06	0.84	1.35	0.49	0.62
Schonberg 2015-1	1.07	0.87	1.32	0.63	0.53
Schonberg 2015-2	1.07	0.89	1.28	0.72	0.47
Rockhill 2001	1.06	0.90	1.25	0.72	0.47
Spiegelman 1994	1.07	0.92	1.25	0.94	0.35
Chlebowski 2007	1.06	0.92	1.22	0.78	0.44
Schonfeld 2010-1	1.05	0.93	1.18	0.76	0.45
Tice 2008	1.04	0.93	1.16	0.71	0.48
	1.04	0.93	1.16	0.71	0.48

Cumulative statistics

Cumulative E/O ratio (95% CI)

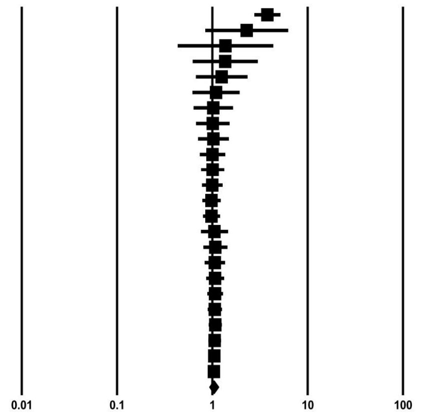

Additional file 6. Forest plot (A), sensitivity analysis (B) and cumulative analysis ranked by publication year (C) and sample size (D) of the calibration of the Gail model after excluding the studies conducted in Asian women.
