# Supplementary material for: Assessment of performance of the Gail model for predicting breast cancer risk: a systematic review and meta-analysis with trial sequential analysis
Source: Breast Cancer Res. 2018 Mar 13;20:18. doi: 10.1186/s13058-018-0947-5 (PMC5850919; doi:10.1186/s13058-018-0947-5)
Supplement: Supplementary file 7 — Shows pooled E/O ratio for Gail model 1 and Caucasian-American Gail model 2 after excluding studies conducted in Asian women. (PDF 582 kb) [file 13058_2018_947_MOESM7_ESM.pdf]

Meta Analysis

Study name	Subgroup within study	E/O ratio	Lower limit	Upper limit	Z-Value	p-Value
Costantino 1999-2	Caucasian-American model	1.03	0.88	1.21	0.36	0.72
Rockhill 2001	Caucasian-American model	0.94	0.89	0.99	-2.28	0.02
Amir 2003	Caucasian-American model	0.69	0.53	0.89	-2.85	0.00
Boyle 2004	Caucasian-American model	1.16	0.90	1.50	1.13	0.26
Decarli 2006	Caucasian-American model	0.93	0.81	1.07	-1.02	0.31
Chlebowski 2007	Caucasian-American model	0.79	0.77	0.81	-18.25	0.00
Tice 2008	Caucasian-American model	0.88	0.86	0.90	-11.02	0.00
Schonfeld 2010-1	Caucasian-American model	0.87	0.85	0.89	-11.87	0.00
Schonfeld 2010-2	Caucasian-American model	0.86	0.82	0.90	-6.35	0.00
Tarabishy 2011	Caucasian-American model	0.93	0.75	1.15	-0.68	0.50
Pastor-Barriuso 2013	Caucasian-American model	1.46	1.36	1.56	10.81	0.00
Buron 2013	Caucasian-American model	0.58	0.54	0.62	-15.46	0.00
Powell 2014	Caucasian-American model	0.81	0.71	0.93	-3.06	0.00
McCarthy 2015	Caucasian-American model	3.78	2.78	5.13	8.51	0.00
Dartois 2015	Caucasian-American model	0.97	0.84	1.12	-0.42	0.68
Brentnall 2015	Caucasian-American model	2.67	2.46	2.90	23.40	0.00
Schonberg 2015-1	Caucasian-American model	1.20	1.13	1.27	6.12	0.00
Schonberg 2015-2	Caucasian-American model	1.05	1.00	1.10	2.01	0.04
	**Combined**	1.05	0.93	1.17	0.77	0.44
Bondy 1994	Gail model 1	1.31	0.96	1.79	1.70	0.09
Spiegelman 1994	Gail model 1	1.33	1.28	1.38	14.86	0.00
Costantino 1999-1	Gail model 1	0.84	0.73	0.97	-2.40	0.02
Bernatsky 2004	Gail model 1	0.48	0.29	0.80	-2.84	0.00
Olson 2004	Gail model 1	1.37	0.92	2.04	1.55	0.12
	**Combined**	1.03	0.76	1.40	0.19	0.85
	**Overall combined**	1.04	0.94	1.16	0.79	0.43

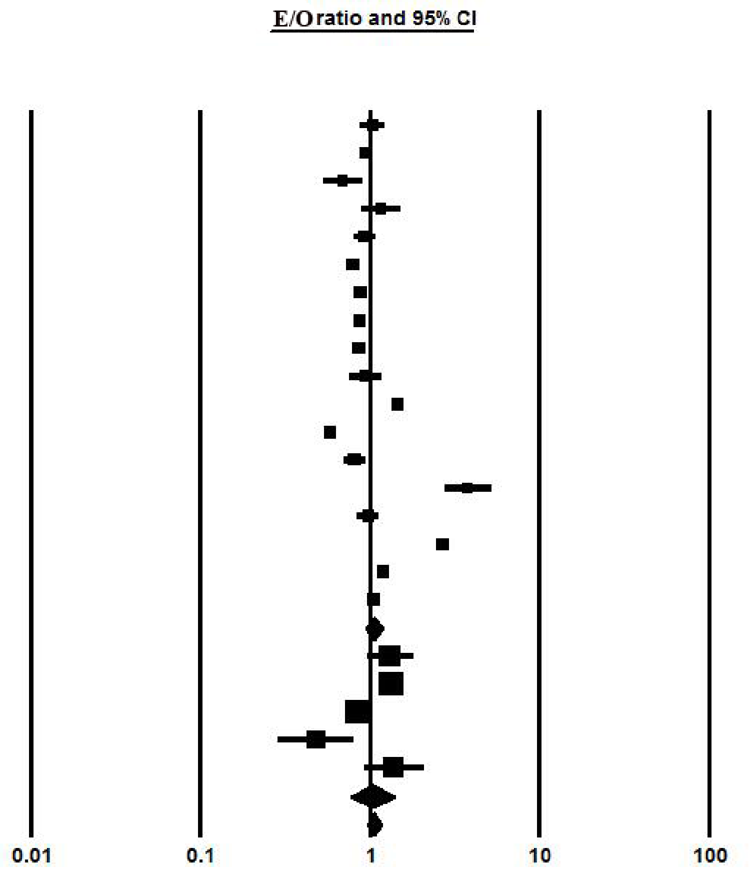

Meta Analysis

Additional file 7. The pooled E/O ratio for the Gail model 1 and Caucasian-American Gail model after excluding the studies conducted in Asian women.
