# Supplementary material for: Assessment of performance of the Gail model for predicting breast cancer risk: a systematic review and meta-analysis with trial sequential analysis
Source: Breast Cancer Res. 2018 Mar 13;20:18. doi: 10.1186/s13058-018-0947-5 (PMC5850919; doi:10.1186/s13058-018-0947-5)
Supplement: Supplementary file 8 — Shows subgroup analysis of calibration of the Gail model after excluding studies conducted in Asian women. (PDF 106 kb) [file 13058_2018_947_MOESM8_ESM.pdf]

Subgroup	No. of studies	E/O ratio (95%CI)	p-value
Geographic background			0.89
America	16	1.02(0.93-1.12)	
Europe and others	8	1.05(0.68-1.63)	
Sample size			0.70
≥10000	13	1.07(0.94-1.22)	
<10000	11	1.01(0.77-1.32)	
Study quality			0.06
≥8 points	8	0.88(0.71-1.10)	
<8 points	16	1.13(1.00-1.29)	
Risk prediction period			0.66
5 years	21	1.01(0.92-1.11)	
10 years	2	1.37(0.36-5.14)	
Type of Gail model			0.93
Gail 1	5	1.03(0.76-1.40)	
Gail 2	18	1.05(0.93-1.17)	
Study period			0.15
Before 2000 year	17	0.97(0.88-1.07)	
After 2000 year	6	1.05(0.93-1.17)	
Population type			0.54
General population	15	1.03(0.90-1.18)	
High-risk population	8	1.11(0.93-1.32)	
**Overall**	**24**	**1.04(0.94-1.16)**	

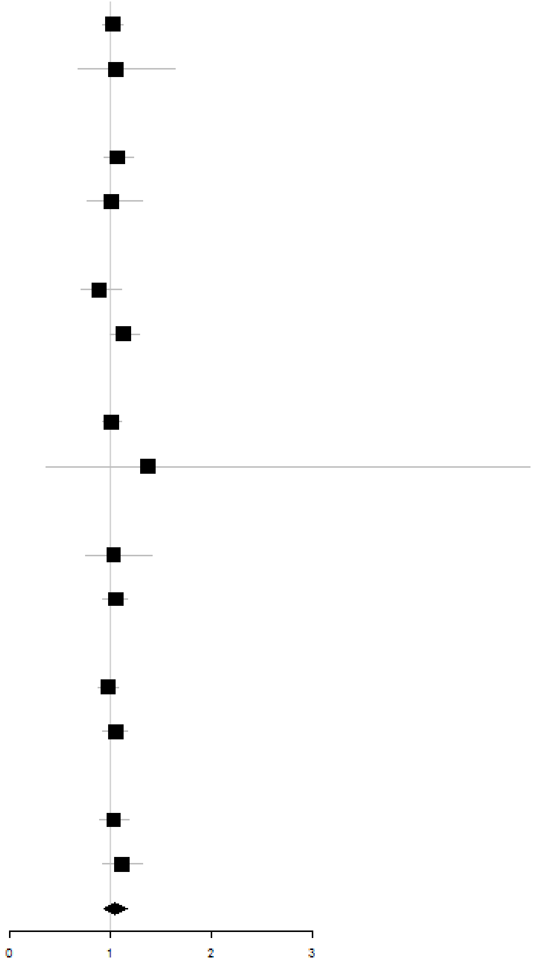

Additional file 8. Subgroup analysis of the calibration of the Gail model after excluding the studies conducted in Asian women.
