# Supplementary material for: Assessment of performance of the Gail model for predicting breast cancer risk: a systematic review and meta-analysis with trial sequential analysis
Source: Breast Cancer Res. 2018 Mar 13;20:18. doi: 10.1186/s13058-018-0947-5 (PMC5850919; doi:10.1186/s13058-018-0947-5)
Supplement: Supplementary file 9 — Shows sensitivity analysis (A), cumulative meta-analysis ranked by publication year (B) and sample size (C) of discrimination of the Gail model. (PDF 1321 kb) [file 13058_2018_947_MOESM9_ESM.pdf]

A

Study name	AUC	Standard error	Variance	Lower limit	Upper limit	Z-Value	p-Value
	Statistics with study removed						
Rockhill 2001	0.60	0.01	0.00	0.58	0.63	46.27	0.00
Amir 2003	0.60	0.01	0.00	0.57	0.62	47.18	0.00
Tice 2005	0.60	0.01	0.00	0.57	0.62	46.85	0.00
Decarli 2006	0.60	0.01	0.00	0.58	0.63	46.91	0.00
Crispo 2008	0.60	0.01	0.00	0.58	0.63	46.93	0.00
Tice 2008	0.60	0.01	0.00	0.57	0.63	43.41	0.00
Pan 2009	0.60	0.01	0.00	0.57	0.62	46.64	0.00
Liu 2010	0.60	0.01	0.00	0.58	0.63	47.30	0.00
Wang 2010	0.59	0.01	0.00	0.57	0.61	53.44	0.00
Tarabishy 2011	0.60	0.01	0.00	0.57	0.62	46.22	0.00
Vacek 2011	0.60	0.01	0.00	0.58	0.63	47.26	0.00
Banegas 2012	0.60	0.01	0.00	0.57	0.63	44.13	0.00
Quante 2012	0.60	0.01	0.00	0.57	0.62	46.94	0.00
Pastor-Barriuso 2013	0.60	0.01	0.00	0.58	0.63	47.26	0.00
Dite 2013	0.60	0.01	0.00	0.58	0.63	46.76	0.00
Anothaisintawee 2013	0.61	0.01	0.00	0.58	0.63	48.98	0.00
Ronser 2013	0.60	0.01	0.00	0.58	0.63	46.93	0.00
Min 2014-1	0.60	0.01	0.00	0.58	0.63	47.24	0.00
Min 2014-2	0.60	0.01	0.00	0.58	0.63	47.28	0.00
Powell 2014	0.60	0.01	0.00	0.57	0.62	46.60	0.00
Duan 2014	0.60	0.01	0.00	0.58	0.63	47.31	0.00
McCarthy 2015	0.60	0.01	0.00	0.57	0.62	47.10	0.00
Dartois 2015-1	0.60	0.01	0.00	0.58	0.63	47.07	0.00
Dartois 2015-2	0.60	0.01	0.00	0.58	0.63	47.24	0.00
Hu 2015	0.60	0.01	0.00	0.58	0.63	47.44	0.00
Brentnall 2015	0.60	0.01	0.00	0.58	0.63	47.26	0.00
Schonberg 2015-1	0.60	0.01	0.00	0.58	0.63	46.44	0.00
Schonberg 2015-2	0.60	0.01	0.00	0.58	0.63	46.27	0.00
Rong 2016	0.60	0.01	0.00	0.57	0.62	52.87	0.00
	0.60	0.01	0.00	0.58	0.62	47.98	0.00

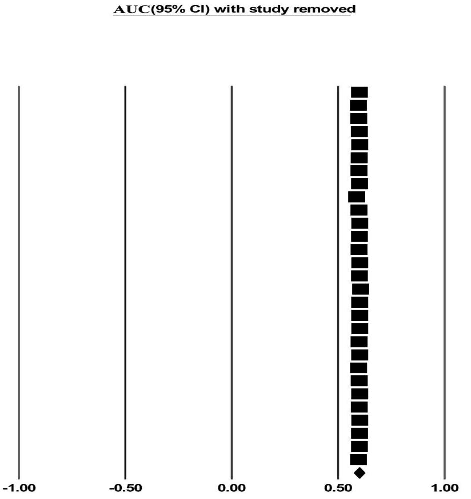

Meta Analysis

B

Study name	Publication year	AUC	Standard error	Variance	Lower limit	Upper limit	Z-Value	p-Value
		Cumulative statistics						
Rockhill 2001	2001	0.58	0.01	0.00	0.56	0.60	56.84	0.00
Amir 2003	2003	0.66	0.08	0.01	0.50	0.81	8.21	0.00
Tice 2005	2005	0.66	0.04	0.00	0.58	0.74	16.44	0.00
Decarli 2006	2006	0.64	0.03	0.00	0.58	0.70	20.31	0.00
Crispo 2008	2008	0.62	0.03	0.00	0.56	0.68	21.72	0.00
Tice 2008	2008	0.62	0.02	0.00	0.58	0.65	32.41	0.00
Pan 2009	2009	0.62	0.02	0.00	0.59	0.65	36.41	0.00
Liu 2010	2010	0.61	0.02	0.00	0.58	0.65	37.96	0.00
Wang 2010	2010	0.65	0.03	0.00	0.60	0.70	24.29	0.00
Tarabishy 2011	2011	0.65	0.02	0.00	0.60	0.70	27.80	0.00
Vacek 2011	2011	0.64	0.02	0.00	0.60	0.68	28.75	0.00
Banegas 2012	2012	0.63	0.02	0.00	0.60	0.67	34.19	0.00
Quante 2012	2012	0.63	0.02	0.00	0.60	0.67	35.74	0.00
Pastor-Barriuso 2013	2013	0.63	0.02	0.00	0.59	0.66	36.92	0.00
Dite 2013	2013	0.62	0.02	0.00	0.59	0.65	38.71	0.00
Anothaisintawee 2013	2013	0.61	0.02	0.00	0.58	0.64	37.38	0.00
Ronser 2013	2013	0.61	0.02	0.00	0.58	0.64	39.10	0.00
Min 2014-1	2014	0.60	0.02	0.00	0.57	0.63	40.11	0.00
Min 2014-2	2014	0.60	0.01	0.00	0.57	0.63	41.11	0.00
Powell 2014	2014	0.60	0.01	0.00	0.57	0.63	42.83	0.00
Duan 2014	2014	0.60	0.01	0.00	0.57	0.63	43.72	0.00
McCarthy 2015	2015	0.60	0.01	0.00	0.58	0.63	44.60	0.00
Dartois 2015-1	2015	0.60	0.01	0.00	0.58	0.63	45.69	0.00
Dartois 2015-2	2015	0.60	0.01	0.00	0.58	0.63	46.61	0.00
Hu 2015	2015	0.60	0.01	0.00	0.58	0.63	47.22	0.00
Brentnall 2015	2015	0.60	0.01	0.00	0.57	0.62	48.46	0.00
Schonberg 2015-1	2015	0.60	0.01	0.00	0.57	0.62	50.59	0.00
Schonberg 2015-2	2015	0.60	0.01	0.00	0.57	0.62	52.87	0.00
Rong 2016	2016	0.60	0.01	0.00	0.58	0.62	47.98	0.00
		0.60	0.01	0.00	0.58	0.62	47.98	0.00

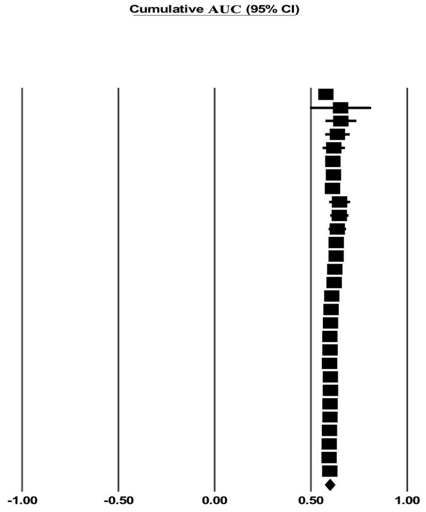

Meta Analysis

C

Study name	Publication year	AUC	Standard error	Variance	Lower limit	Upper limit	Z-Value	p-Value
		Cumulative statistics						
Wang 2010	228	0.93	0.02	0.00	0.89	0.97	45.81	0.00
Liu 2010	246	0.75	0.18	0.03	0.38	1.11	4.03	0.00
Duan 2014	400	0.68	0.14	0.02	0.40	0.96	4.77	0.00
McCarthy 2015	464	0.69	0.10	0.01	0.48	0.89	6.63	0.00
Rong 2016	816	0.69	0.06	0.00	0.57	0.80	11.54	0.00
Dite 2013	1425	0.67	0.05	0.00	0.57	0.76	13.89	0.00
Crispo 2008	1765	0.65	0.04	0.00	0.57	0.74	15.17	0.00
Quante 2012	1857	0.65	0.04	0.00	0.57	0.73	16.67	0.00
Pan 2009	2133	0.65	0.03	0.00	0.58	0.71	19.08	0.00
Amir 2003	3150	0.66	0.03	0.00	0.59	0.72	20.47	0.00
Tarabishy 2011	4726	0.66	0.03	0.00	0.60	0.71	24.14	0.00
Dartois 2015-1	5843	0.65	0.03	0.00	0.60	0.70	25.28	0.00
Dartois 2015-2	7331	0.64	0.02	0.00	0.60	0.69	25.93	0.00
Decarli 2006	10031	0.64	0.02	0.00	0.59	0.69	27.15	0.00
Ronser 2013	11419	0.63	0.02	0.00	0.59	0.68	27.54	0.00
Powell 2014	12843	0.63	0.02	0.00	0.59	0.67	29.36	0.00
Anothaisintawee 2013	15718	0.62	0.02	0.00	0.58	0.66	27.99	0.00
Vacek 2011	19779	0.62	0.02	0.00	0.57	0.66	28.60	0.00
Min 2014-1	40229	0.61	0.02	0.00	0.57	0.65	29.32	0.00
Min 2014-2	40229	0.61	0.02	0.00	0.57	0.65	30.00	0.00
Hu 2015	42908	0.61	0.02	0.00	0.57	0.65	30.58	0.00
Brentnall 2015	50628	0.60	0.02	0.00	0.57	0.64	31.45	0.00
Pastor-Barriuso 2013	54649	0.60	0.02	0.00	0.57	0.64	32.45	0.00
Schonberg 2015	71293	0.60	0.02	0.00	0.57	0.63	34.18	0.00
Schonberg 2015	79611	0.60	0.02	0.00	0.57	0.63	36.05	0.00
Tice 2005	81777	0.60	0.02	0.00	0.57	0.63	37.58	0.00
Rockhill 2001	82109	0.60	0.02	0.00	0.57	0.63	39.34	0.00
Banegas 2012	128976	0.60	0.01	0.00	0.57	0.63	43.41	0.00
Tice 2008	251789	0.60	0.01	0.00	0.58	0.62	47.98	0.00
		0.60	0.01	0.00	0.58	0.62	47.98	0.00

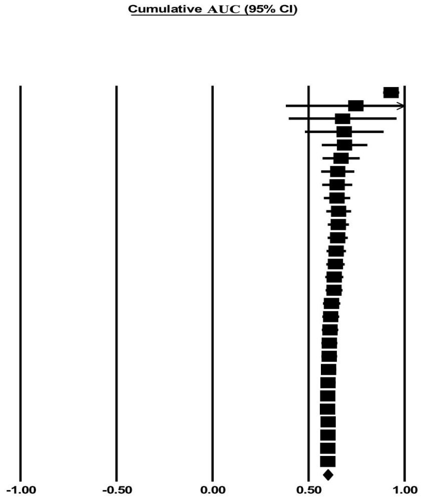

Meta Analysis

Additional file 9. The sensitivity analysis (A), cumulative meta-analysis ranked by the publication year (B) and sample size (C) of the discrimination of the Gail model.
