# Supplementary material for: Assessment of performance of the Gail model for predicting breast cancer risk: a systematic review and meta-analysis with trial sequential analysis
Source: Breast Cancer Res. 2018 Mar 13;20:18. doi: 10.1186/s13058-018-0947-5 (PMC5850919; doi:10.1186/s13058-018-0947-5)
Supplement: Supplementary file 11 — Shows funnel plot of discrimination of the Gail model (A) and funnel plot adjusted by trim-and-fill method (B). (PDF 226 kb) [file 13058_2018_947_MOESM11_ESM.pdf]

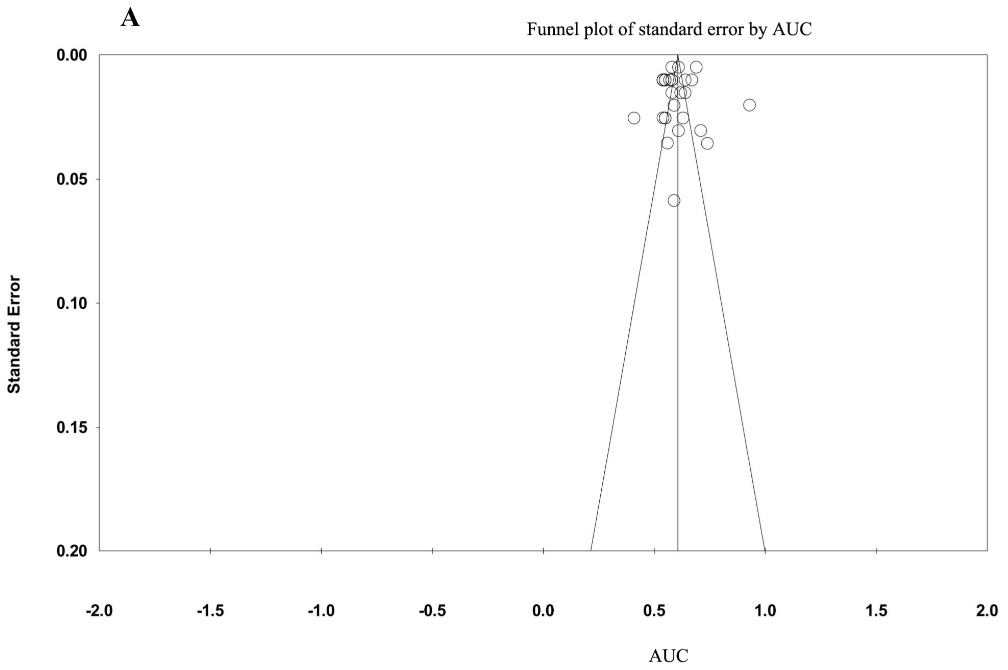

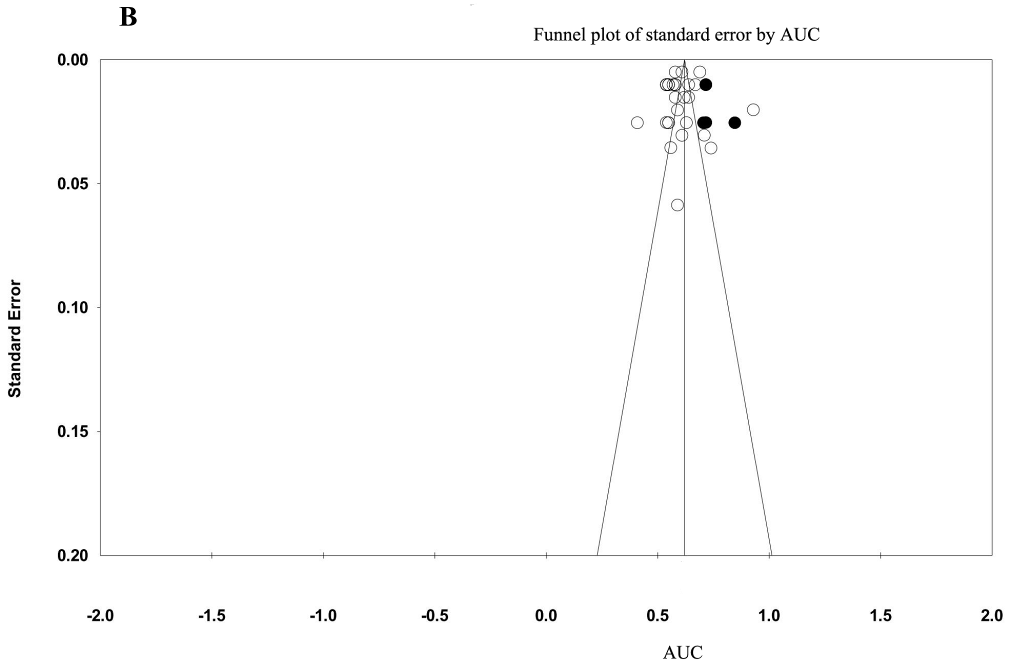

Additional file 11. Funnel plot of the discrimination of the Gail model (A) and the funnel plot adjusted by trim-and-fill method (B).
