# Supplementary material for: Assessment of performance of the Gail model for predicting breast cancer risk: a systematic review and meta-analysis with trial sequential analysis
Source: Breast Cancer Res. 2018 Mar 13;20:18. doi: 10.1186/s13058-018-0947-5 (PMC5850919; doi:10.1186/s13058-018-0947-5)
Supplement: Supplementary file 13 — Shows pooled AUC for Caucasian-American Gail model 2 in American, Asian and European women and Gail model 1 in American and European women and Asian females. (PDF 686 kb) [file 13058_2018_947_MOESM13_ESM.pdf]

Meta Analysis

Study name	Subgroup within study	AUC	Standard error	Variance	Lower limit	Upper limit	Z-Value	p-Value
Rockhill 2001	Caucasian-American model for American	0.58	0.01	0.00	0.56	0.60	56.84	0.00
Tice 2005	Caucasian-American model for American	0.67	0.01	0.00	0.65	0.69	65.66	0.00
Tice 2008	Caucasian-American model for American	0.61	0.01	0.00	0.60	0.62	119.56	0.00
Tarabishy 2011	Caucasian-American model for American	0.64	0.01	0.00	0.62	0.66	62.73	0.00
Banegas 2012	Caucasian-American model for American	0.58	0.01	0.00	0.57	0.59	113.68	0.00
Quante 2012	Caucasian-American model for American	0.63	0.03	0.00	0.58	0.68	24.71	0.00
Ronser 2013	Caucasian-American model for American	0.55	0.01	0.00	0.53	0.57	53.90	0.00
Powell 2014	Caucasian-American model for American	0.62	0.02	0.00	0.59	0.65	40.51	0.00
McCarthy 2015	Caucasian-American model for American	0.71	0.03	0.00	0.65	0.77	23.25	0.00
Schonberg 2015-1	Caucasian-American model for American	0.57	0.01	0.00	0.55	0.59	55.86	0.00
Schonberg 2015-2	Caucasian-American model for American	0.58	0.01	0.00	0.56	0.60	56.84	0.00
	**Combined**	0.61	0.01	0.00	0.59	0.63	56.36	0.00
Liu 2010	Caucasian-American model for Asian	0.56	0.04	0.00	0.49	0.63	15.76	0.00
Min 2014-1	Caucasian-American model for Asian	0.55	0.03	0.00	0.50	0.60	21.56	0.00
Duan 2014	Caucasian-American model for Asian	0.54	0.03	0.00	0.49	0.59	21.23	0.00
Hu 2015	Caucasian-American model for Asian	0.59	0.06	0.00	0.48	0.70	10.06	0.00
	**Combined**	0.55	0.02	0.00	0.52	0.58	35.56	0.00
Amir 2003	Caucasian-American model for European	0.74	0.04	0.00	0.67	0.81	20.73	0.00
Decarli 2006	Caucasian-American model for European	0.59	0.02	0.00	0.55	0.63	28.91	0.00
Pastor-Barriuso 2013	Caucasian-American model for European	0.54	0.01	0.00	0.52	0.56	52.92	0.00
Dite 2013	Caucasian-American model for European	0.58	0.02	0.00	0.55	0.61	37.92	0.00
Dartois 2015-1	Caucasian-American model for European	0.61	0.03	0.00	0.55	0.67	19.93	0.00
Dartois 2015-2	Caucasian-American model for European	0.55	0.03	0.00	0.50	0.60	21.56	0.00
Brentnall 2015	Caucasian-American model for European	0.54	0.01	0.00	0.52	0.56	52.92	0.00
	**Combined**	0.58	0.02	0.00	0.55	0.62	34.61	0.00
Crispo 2008	Gail model 1 for American and European	0.55	0.01	0.00	0.53	0.57	53.94	0.00
Vacek 2011	Gail model 1 for American and European	0.54	0.01	0.00	0.52	0.56	52.92	0.00
	**Combined**	0.55	0.01	0.00	0.53	0.56	75.56	0.00
Pan 2009	Gail model 1 for Asian	0.64	0.02	0.00	0.61	0.67	41.84	0.00
Wang 2010	Gail model 1 for Asian	0.93	0.02	0.00	0.89	0.97	45.81	0.00
Rong 2016	Gail model 1 for Asian	0.69	0.01	0.00	0.68	0.70	135.44	0.00
	**Combined**	0.75	0.06	0.00	0.63	0.88	11.67	0.00
	**Overall combined**	0.57	0.01	0.00	0.56	0.58	107.01	0.00

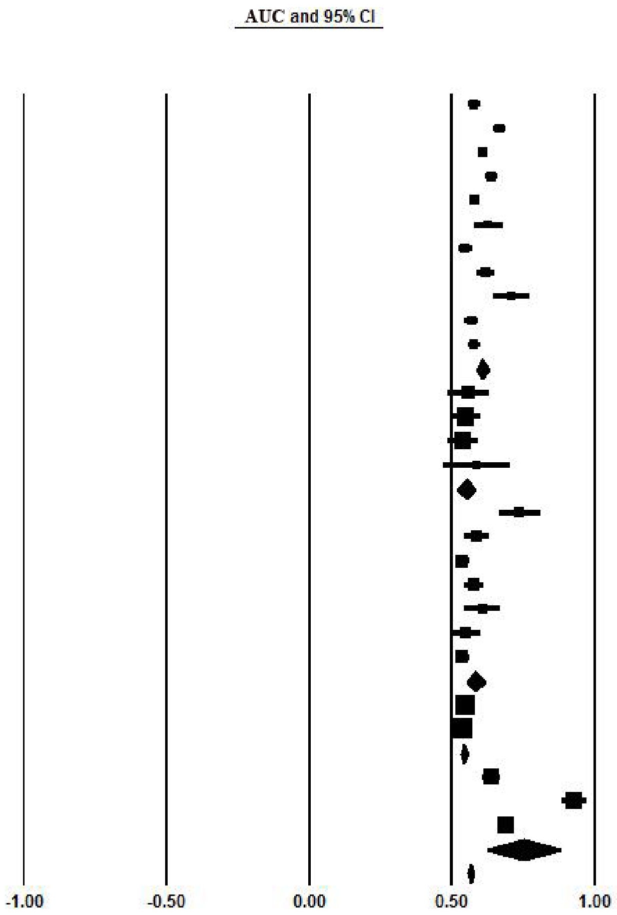

Meta Analysis

Additional file 13. The pooled AUC for the Caucasian-American Gail model 2 in American, Asian and European women and the Gail model 1 in American and European women and Asian females.
