# Supplementary material for: Assessment of performance of the Gail model for predicting breast cancer risk: a systematic review and meta-analysis with trial sequential analysis
Source: Breast Cancer Res. 2018 Mar 13;20:18. doi: 10.1186/s13058-018-0947-5 (PMC5850919; doi:10.1186/s13058-018-0947-5)
Supplement: Supplementary file 14 — Shows Deeks’ funnel plot of diagnostic accuracy meta-analysis (A) and funnel plot of stratified analysis in America and Europe (B) and Asia (C). (PDF 294 kb) [file 13058_2018_947_MOESM14_ESM.pdf]

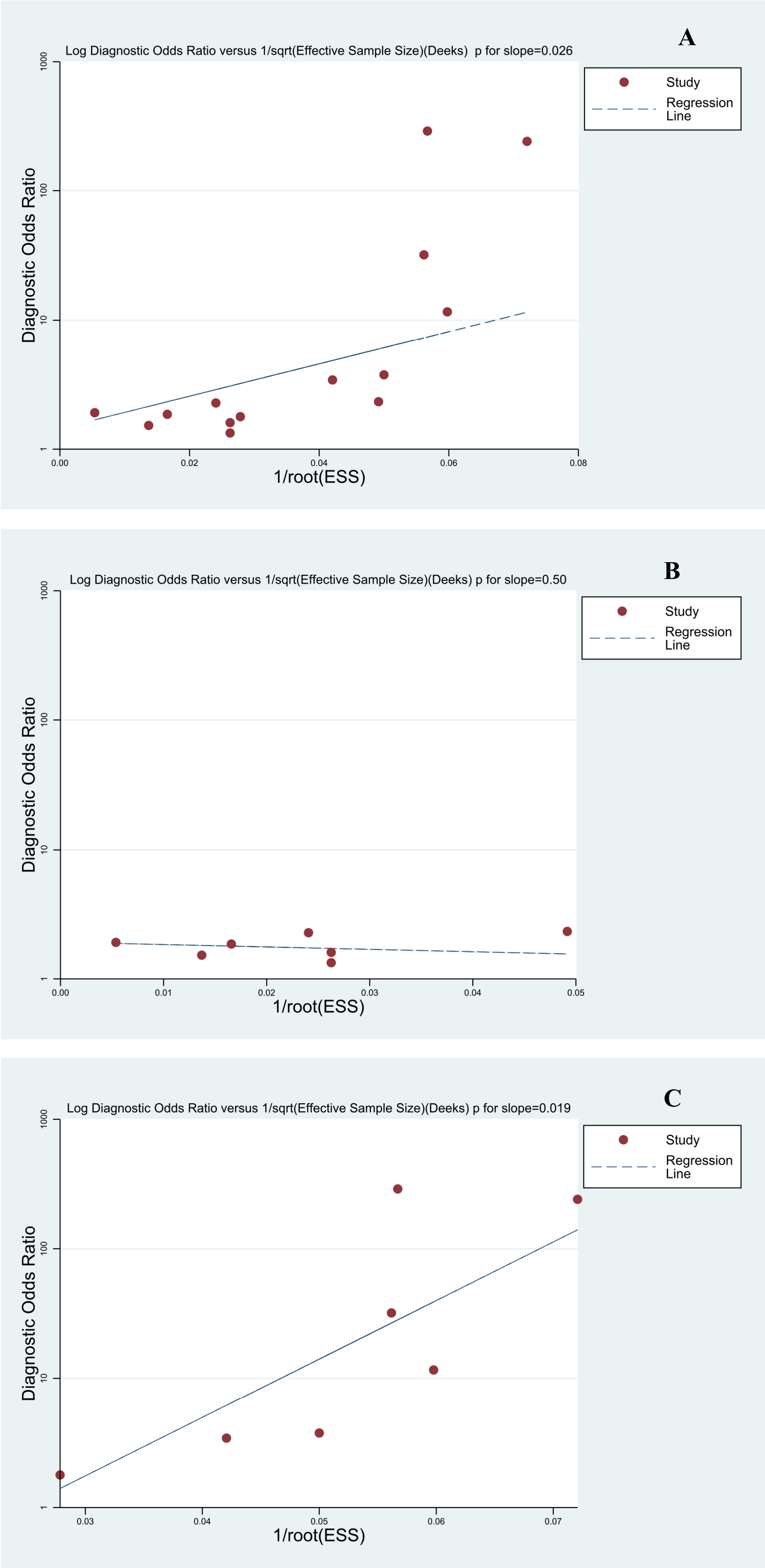

Additional file 14. Deek's funnel plot of diagnostic accuracy meta-analysis (A) and the funnel plot in the stratified analysis in America and Europe (B) and Asia (C).
