# Supplementary material for: Assessment of performance of the Gail model for predicting breast cancer risk: a systematic review and meta-analysis with trial sequential analysis
Source: Breast Cancer Res. 2018 Mar 13;20:18. doi: 10.1186/s13058-018-0947-5 (PMC5850919; doi:10.1186/s13058-018-0947-5)
Supplement: Supplementary file 16 — Shows pooled sensitivity, specificity and DOR of the Caucasian-American Gail model in American and European women (A) and Asian females (B). (PDF 828 kb) [file 13058_2018_947_MOESM16_ESM.pdf]

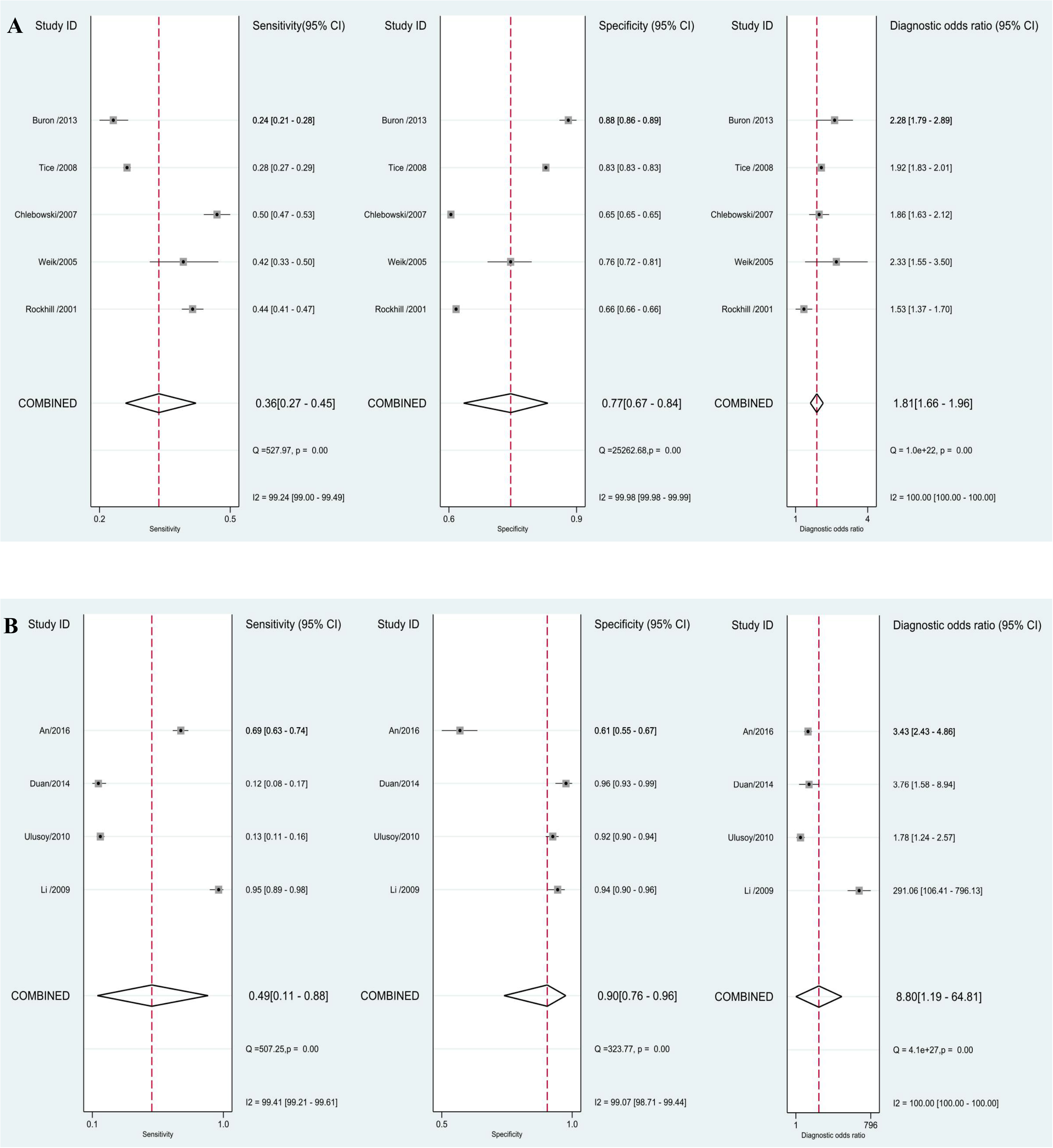

Additional file 16. The pooled sensitivity, specificity and diagnostic odds ratio of the Caucasian-American Gail model in American and European women (A) and Asian females(B).
