# Supplementary material for: Assessment of performance of the Gail model for predicting breast cancer risk: a systematic review and meta-analysis with trial sequential analysis
Source: Breast Cancer Res. 2018 Mar 13;20:18. doi: 10.1186/s13058-018-0947-5 (PMC5850919; doi:10.1186/s13058-018-0947-5)
Supplement: Supplementary file 18 — Shows calibration (A) and discrimination (B) of different versions of the Gail model after excluding studies published in Chinese. (PDF 531 kb) [file 13058_2018_947_MOESM18_ESM.pdf]

A

Meta Analysis

Study name	Subgroup within study	E/O ratio	Lower limit	Upper limit	Z-Value	p-Value
Chay 2012-1	Asian-American Gail model	2.51	2.13	2.95	11.12	0.00
Chay 2012-2	Asian-American Gail model	1.85	1.68	2.04	12.42	0.00
Min 2014-2	Asian-American Gail model	1.29	1.11	1.50	3.24	0.00
	**Combined**	1.82	1.31	2.51	3.59	0.00
Min 2014-1	Caucasian-American Gail model	2.46	2.10	2.88	11.30	0.00
	**Combined**	2.46	2.10	2.88	11.30	0.00
	**Overall Combined**	2.32	2.02	2.68	11.74	0.00

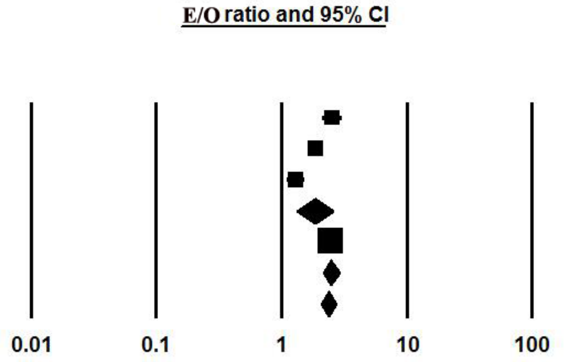

Meta Analysis

B

Meta Analysis

Study name	Subgroup within study	AUC	Standard error	Variance	Lower limit	Upper limit	Z-Value	p-Value
Min 2014-2	Asian-American model for Asia	0.54	0.02	0.00	0.50	0.58	26.46	0.00
	**Combined**	0.54	0.02	0.00	0.50	0.58	26.46	0.00
Min 2014-1	Caucasian-American model for Asia	0.55	0.03	0.00	0.50	0.60	21.56	0.00
	**Combined**	0.55	0.03	0.00	0.50	0.60	21.56	0.00
Vacek 2011	Gail model 1	0.54	0.01	0.00	0.52	0.56	52.92	0.00
Crispo 2008	Gail model 1	0.55	0.01	0.00	0.53	0.57	53.94	0.00
	**Combined**	0.55	0.01	0.00	0.53	0.56	75.56	0.00
	**Overall combined**	0.54	0.01	0.00	0.53	0.56	82.91	0.00

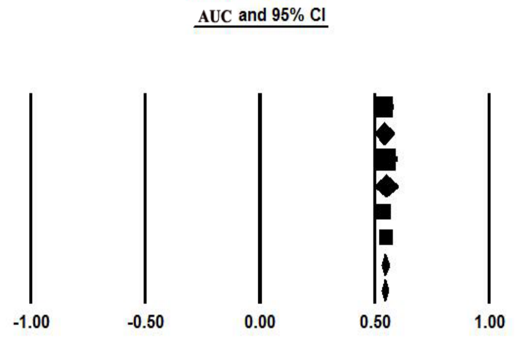

Meta Analysis

Additional file 18. The calibration (A) and discrimination (B) of different versions of the Gail model after excluding the studies published in Chinese.
